# Safety and Tolerability of Pivmecillinam During More Than Four Decades of Clinical Experience: A Systematic Review

**DOI:** 10.1093/cid/ciae621

**Published:** 2025-01-21

**Authors:** Keith S Kaye, Anne Santerre Henriksen, Morten Sommer, Niels Frimodt-Møller

**Affiliations:** Division of Allergy, Immunology and Infectious Diseases, Rutgers Robert Wood Johnson Medical School, New Brunswick, New Jersey, USA; Clinical Development, UTILITY therapeutics, London, United Kingdom; Clinical Development, Maxel Consulting ApS, Jyllinge, Denmark; Clinical Development, UTILITY therapeutics, London, United Kingdom; DTU Biosustain, Technical University of Denmark, Lyngby, Denmark; Department of Clinical Microbiology, Rigshospitalet, Copenhagen, Denmark

**Keywords:** antibiotic, mecillinam, pivmecillinam, safety, urinary tract infection

## Abstract

The recent US Food and Drug Administration approval of pivmecillinam—an oral prodrug of the amidinopenicillin antibiotic mecillinam—presents a valuable opportunity to address the need for new treatments for uncomplicated urinary tract infection (uUTI). We report findings of a systematic literature review of the safety profile of pivmecillinam/mecillinam based on more than 40 years’ experience, mainly in Europe and Canada, to describe its tolerability profile and identify any important safety signals. In total, 110 eligible publications were identified describing use of pivmecillinam/mecillinam as monotherapy or in combination, for treatment of uUTI or other infectious conditions. These studies revealed a benign safety and tolerability profile, awareness of which will inform treatment decisions as pivmecillinam is made available in the United States. Together with the evidence for efficacy of, and minimal resistance to, pivmecillinam, the findings of this review support the position of pivmecillinam as a first-line treatment for uUTI.

## Teaser

Pivmecillinam has recently been approved for treatment of uncomplicated urinary tract infection in the United States. This systematic review of 110 studies spanning more than 4 decades revealed a benign safety and tolerability profile with very few serious adverse events reported.

In April 2024, pivmecillinam became the first antibiotic in approximately 20 years to receive US Food and Drug Administration (FDA) approval for uncomplicated urinary tract infection (uUTI) [1, 2]. It has been used elsewhere—mainly in Europe and Canada—for over 40 years [3–6], and key guidelines recommend pivmecillinam as a first-line treatment for uUTIs [7, 8]. Pivmecillinam is an oral prodrug of mecillinam, an amidinopenicillin antibiotic that binds exclusively to penicillin-binding protein (PBP)-2 in enteric bacteria [3,9]. Mecillinam acts synergistically with other beta-lactams that bind to PBP1a/PBP1b and/or PBP3 in gram-negative bacteria [10] and, when used in combination, may also have the potential to constrain evolution of extended-spectrum beta-lactamases [11]. In Europe, oral pivmecillinam is also indicated for treatment of salmonellosis [4, 5] and recommended in certain regions for treatment of acute pyelonephritis [12]; peak serum levels of mecillinam are reached approximately 1 hour post-dose and the serum half-life is 1.2 hours [4, 5].

There is a need for new antibiotic options for uUTI in the United States [13, 14]. The rate of development and approval of new antibiotics has decreased steadily since the 1980s due to economic and regulatory obstacles [15]. The proportion of infections caused by antibiotic-resistant gram-negative bacteria is increasing in community and healthcare settings [13, 16], such that the value of commonly used agents such as trimethoprim-sulfamethoxazole (TMP-SMX), nitrofurantoin, and fluoroquinolones is being eroded [17, 18].

Treatment choices for uUTI are also influenced by safety considerations, including gastrointestinal (GI) and microbiome-related events, with risks varying by antibiotic agent and duration [19]. Rare but serious allergic skin reactions such as toxic epidermal necrolysis have been reported [20–22], although more specific safety concerns include acute or chronic pulmonary toxicity with nitrofurantoin [23]. Fluoroquinolones have been linked with potentially permanent effects; the US FDA recommends that they are only used for uUTI when no alternative treatment options are available [24–26] and antimicrobial stewardship initiatives seek to reduce inappropriate prescribing of fluoroquinolones [27].

Given the recent US approval of pivmecillinam, it is appropriate to review the safety dataset that has amassed in other countries during clinical experience with pivmecillinam over more than 4 decades.

## METHODS

### Objectives

A systematic review was performed to identify safety signals associated with pivmecillinam/mecillinam as antibiotic therapy (not limited to uUTI setting), and adverse events (AEs) related to pivmecillinam, in the published literature.

### Review Methodology

Review methodology and reporting were based on the Cochrane Handbook for Systematic Reviews of Interventions and the Preferred Reporting Items for Systematic Review and Meta-Analysis (PRISMA) guidelines [28]. Literature was identified using population, intervention, comparator, outcome, and study type (PICOS) criteria (Supplementary Table 1).

### Search Strategy

A broad systematic search was conducted to identify all relevant records published in Embase and MEDLINE (PubMed) from inception to October 2023 (Supplementary Tables 2 and 3).

### Screening

First-pass screening of titles/abstracts was performed to identify eligible publications, following removal of duplicates. Exclusions were confirmed by another analyst. A senior reviewer authenticated the results and resolved any ambiguities.

Retained articles were subjected to a full-text review to assess eligibility. Discrepancies were resolved by a senior reviewer.

### Data Extraction

Relevant data from the included publications were extracted into Excel. For dichotomous outcomes, the number of patients with the event and the number of patients in the treatment arm were extracted. For continuous outcomes, change from baseline in all intervention groups was extracted. For event rates, number of events, number of patients in each treatment arm, and follow-up or exposure time data were extracted. AEs were classified (eg, AE vs serious AE [SAE]) as per the original studies. Dosage information was recorded as published; doses of 200 mg, or multiples thereof, relate to pivmecillinam hydrochloride (200 mg of pivmecillinam hydrochloride is equivalent to 185 mg of pivmecillinam [3]).

## RESULTS

### Study Identification

**Figure 1.**
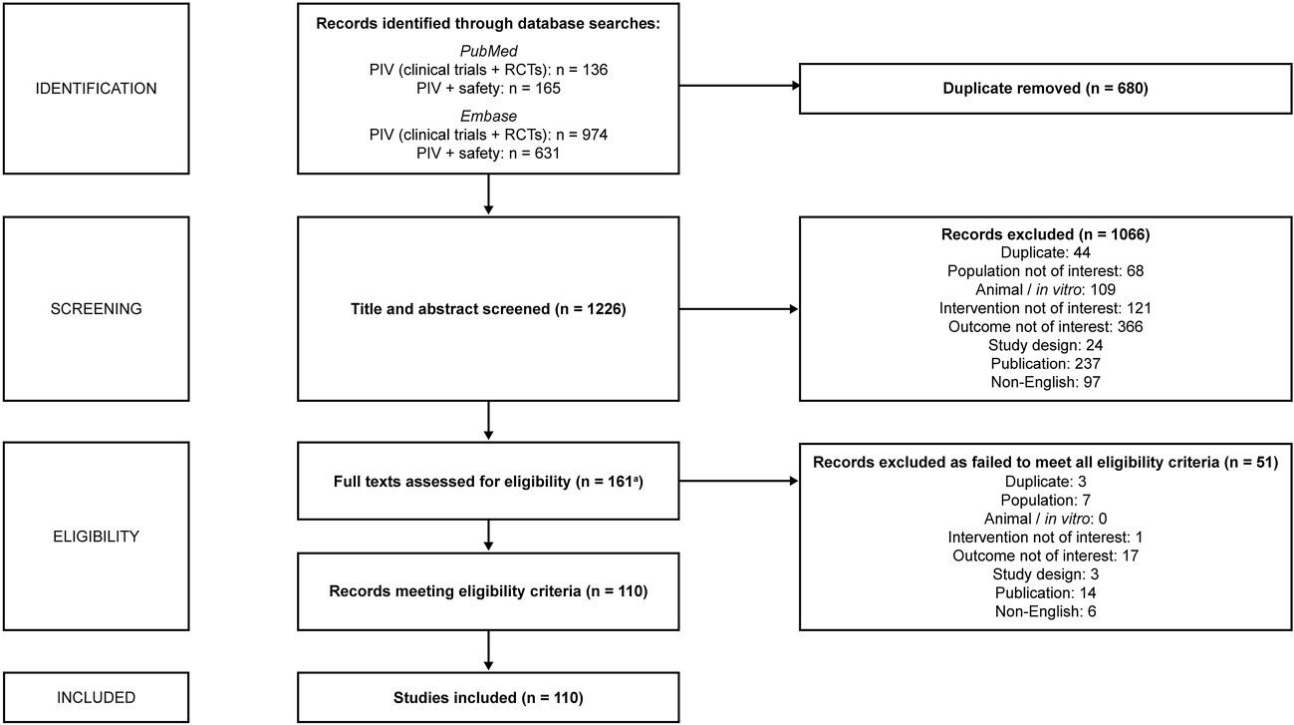
PRISMA diagram. ^a^One reference (Jansåker F, et al EClinicalMedicine, **2019**;12:62–69) was excluded at screening based on lack of safety data in abstract, but subsequently assessed at authors’ discretion, and included [29]. Abbreviations: PIV, pivmecillinam; PRISMA, Preferred Reporting Items for Systematic Reviews and Meta-Analyses; RCT, randomized controlled trial.

In total, 1906 citations were identified (PubMed n = 301, Embase n = 1605; Supplementary Tables 2 and 3). Following screening based on PICOS criteria, 161 references proceeded to full-text review, with 110 included in the final analysis (Figure 1; Supplementary Table 4). Owing to the large quantity of published studies available, and in order to focus attention on identification of any important or unexpected safety issues, studies (n = 47) reporting only mild or no AEs, or that were considered to lack detailed quantitative data on relevant safety outcomes, were summarized only briefly (Supplementary Table 5). The remainder are considered below.

### Safety in Comparison With Other Antibiotics

Nine studies compared mecillinam/pivmecillinam with other antibiotics (Table 1) [30–38].

**Table 1.** Studies Comparing Mecillinam/Pivmecillinam With Other Antibiotics

Study	Design and Study Population	Treatment Groups^a^	Adverse Reactions, n (%)					Discontinued Due to AEs, n (%)
			Overall	GI Tract Related	Skin Related	Genitourinary	Other	
**Pivmecillinam Versus Amoxicillin**								
Ishigami et al, 1977 [30]	Double-blind RCT; Japan Patients with intractable complicated UTI Mostly men aged ≥60 y	PIV 100 mg qid for 5 d (n = 73)	3 (4.1)	Abdominal pain: 1 (1.4) Diarrhea: 1 (1.4) Gastric discomfort: 1 (1.4)	NR	NR	NR	0 (0)
		Amoxicillin 500 mg qid for 5 d (n = 68)	4 (5.9)	Diarrhea: 1 (1.5) Eruption: 1 (1.5) Gastric disturbance: 2 (2.9)	NR	NR	NR	4 (5.9)
Bresky, 1977 [31] ^b^	Prospective cohort; Sweden Patients with symptoms of acute UTI Mostly aged 15–30 y	PIV 400 mg tid for 10 d	23 (19)	Upper GI AEs (nausea, vomiting, anorexia, epigastric pain)	Typical exanthema: 1 Aggravation of pre-existing eczema: 1 Suspected mononucleosis with itching: 1 Mild transient skin reaction to face: 1	NR	NR	7
		Amoxicillin 375 mg tid for 10 d	30 (19)	Lower GI AEs (loose stools, diarrhea)	Skin rash: 9	Vaginitis	NR	6
**Pivmecillinam Versus Norfloxacin**								
Abrahamsson et al, 1995 [32]	RCT; Sweden Adults with acute voiding symptoms Age range 14–79 y	PIV 200 mg tid for 7 d (n = 20)	NR	NR	NR	NR	Serum carnitine level below 11 µmol/L: 7 (35.0) (mean decrease in the reduction of free serum carnitine concentration: 44.6 to 12.9 µmol/L)	NR
		Norfloxacin 200 mg bid for 7 d (n = 24)	NR	NR	NR	NR	Mean free serum carnitine level remained unchanged (40.0 µmol/L before treatment, 40.5 µmol/L after treatment)	NR
Jonsson et al, 1990 [33]	Single-blind RCT; Sweden Patients with signs or symptoms of UTI Age range (for efficacy population) 65–103 y	PIV 200 mg tid for 7 d (n = 170)	6 (3.5)	AEs mostly GI	NR	NR	NR	1 (0.6): nausea and vomiting
		Norfloxacin 200 mg bid for 7 d (n = 168)	2 (1.2)		NR	NR	NR	0 (0)
Nicolle et al, 2002 [34]	Prospective, double-blind RCT; Austria, Belgium, Canada, Denmark, France, Ireland, Netherlands, Switzerland, and UK Women with symptoms of acute uUTI Mean (SD) age 38.5 (13.1) y in PIV group, 38.2 (13.2) y in norfloxacin group	PIV 400 mg bid for 3 d (n = 479)	171 (35.7) 10 (2.1) severe adverse drug reactions	AEs mostly GI	NR	Vaginal candidiasis: 7 (1.5)	NR	4 (0.8)
		Norfloxacin 400 mg bid for 3 d (n = 467)	184 (39.4) 8 (1.7) severe adverse drug reactions	AEs mostly GI	NR	Vaginal candidiasis: 20 (4.3)	NR	10 (2.1)
**Pivmecillinam Versus Other Antibiotics**								
Sattler et al, 1983 [35]	Prospective randomized trial; US Serious gram-negative bacillary infections Age range 25–94 y	MEC 10 mg/kg every 6 h, up to 14 d (n = 38)	8 (21.1)	Diarrhea: 1 (2.6)	NR	NR	Abnormal liver test (SGOT): 4 (10.5) Abnormal liver test (alkaline phosphatase): 3 (7.9)	NR
		Aminoglycoside 1–2 mg/kg every 8 h, up to 14 d (n = 40)	14 (35.0)	Diarrhea: 1 (2.5) Nausea/vomiting: 1 (2.5)	NR	NR	Phlebitis: 1 (2.5) Increased serum creatinine: 6 (15.0) Abnormal liver test (SGOT): 3 (7.5) Abnormal liver test (alkaline phosphatase): 2 (5.0)	NR
Gordin et al, 1987 [36]	RCT; Finland Women with uncomplicated, bacteriologically verified acute lower UTI Age range 17–63 y	PIV 200 mg tid for 3 d (n = 36)	1 (2.8)	NR	Itching on face/palms: 1 (2.8)	Vaginal candidiasis: 1 (2.8)^c^	…	…
		PIV 200 mg tid for 10 d (n = 35)	1 (2.9)	NR	Rash on trunk/hands: 1 (2.9)	Vaginal candidiasis: 2 (5.7)^c^	…	…
		Sulfadiazine 500 mg + trimethoprim 160 mg bid for 3 d (n = 33)	0 (0)	NR	…	…	…	…
		Sulfadiazine 500 mg + trimethoprim 160 mg bid for 10 d (n = 35)	(∼20.0)	Diarrhea: 1 (2.9)	Rash: 2 (5.7) Urticaria: 1 (2.9)	NR	Severe headache: 1 (2.9)	3 (8.6): 2 skin eruptions, 1 headache
Demos and Green, 1983 [37]	Prospective RCT Patients with UTI, urosepsis, and in a variety of other infections Age range 12.9–93.7 y	MEC 10 mg/kg, 4–6 times a day (n = 153)	7 (4.6)^d^	Nausea: 1 (0.7) Vomiting: 1 (0.7) Diarrhea: 1 (0.7)	NR	NR	Drowsiness: 1 (0.7) Lethargy: 1 (0.7) Fever: 1 (0.7) Tenderness at injection site: 1 (0.7) Phlebitis: 3 (2.0) Laboratory test abnormalities: 6 (3.9) (eosinophilia: 2 [1.3], thrombocytopenia: 1 [0.7], thrombocytosis: 1 [0.7], alkaline phosphatase: 1 [0.7], increased SGOT levels: 1 [0.7])	1 (0.7): drug fever and diarrhea
		Tobramycin per manufacturer's instructions (n = 116)	4 (2.7)^e^	NR	NR	NR	Laboratory test abnormalities: 17 (14.7) (neutropenia: 1 [0.9], elevated BUN: 1 [0.9], increased creatinine: 1 [0.9], increased alkaline phosphatase level: 7 [6.0], increased SGOT levels: 4 [3.4], increased bilirubin: 1 [0.9])	NR
		Other agents per manufacturer's instructions (n = 30)^f^		NR	NR	NR	Phlebitis: 1 (3.3); pain at injection site: 1 (3.3); burning at injection site: 1 (3.3); elevated BUN: 1 (3.3)	2 (6.7): rash and phlebitis (both in patients receiving ampicillin)
Menday, 2000 [38]	Double-blind RCT; US Acute uUTI Age range 18–87 y	PIV 200 mg tid for 3 d (n = 219)	13 (5.9)	GI: 11 (5.0) **Severe AEs:** Nausea: 1 (0.5)	Skin and appendage: 2 (0.9)	NR	Central and peripheral nervous system: 1 (0.5) Vision: 1 (0.5) Vascular: 1 (0.5) Resistance mechanism: 1 (0.5)	4 (1.8): esophagitis, nausea, nausea and vomiting, nausea and flushing
		Cephalexin 250 mg qid for 7 d (n = 221)	16 (7.2)	GI: 5 (2.3)	Skin and appendage: 7 (3.2)	**Severe AEs:** Moniliasis: 1 (0.5)	Respiratory: 1 (0.5) Central and peripheral nervous system: 3 (1.4) Psychiatric: 2 (0.9) Musculoskeletal: 1 (0.5) Resistance mechanism: 4 (1.8) Body as a whole: 1 (0.5)	4 (1.8): sweating, vertigo, anxiety and hot flushes, dizziness and diarrhea, urticaria and pruritus

Abbreviations: AE, adverse event; bid, twice daily; BUN, blood urea nitrogen; d, days; GI, gastrointestinal; h, hours; MEC, mecillinam; NR, not reported; PIV, pivmecillinam; qid, 4 times daily; RCT, randomized controlled trial; SD, standard deviation; SGOT, serum glutamic-oxaloacetic transaminase; tid, 3 times daily; UTI, urinary tract infection; uUTI, uncomplicated urinary tract infection; y, years.

^a^n represents size of safety population.

^b^Some n numbers and percentage data are missing from this entry as size of the safety population is unclear. Note that other details from this study are included in Table 2.

^c^Vaginal candidiasis was not included in the total AE count in this report.

^d^Excluding laboratory test abnormalities, which were counted separately.

^e^Note, value applies for “Tobramycin per manufacturer's instructions (n = 116)” and “Other agents per manufacturer's instructions (n = 30)” grouped together.

^f^This group included patients treated with cefazolin (n = 9), ampicillin (n = 8), gentamicin (n = 5), cefamandole (n = 3), carbenicillin (n = 2), cefoxitin (n = 1), chloramphenicol (n = 1), and sulfamethoxazole (n = 1).

Two studies compared pivmecillinam with amoxicillin: a Japanese double-blind randomized controlled trial (RCT) of 148 patients with intractable UTI with complications (mainly men aged ≥60 years) [30] and a Swedish prospective cohort study of 298 outpatients with acute UTI [31]. AE rates were similar, although upper GI effects were more common with pivmecillinam and lower GI effects with amoxicillin.

Three studies compared pivmecillinam with norfloxacin. A Swedish RCT in adult patients with acute voiding symptoms explored treatment effects on serum carnitine concentration, which decreased in 35% of pivmecillinam-treated patients but in none treated with norfloxacin [32]. A single-blind RCT of hospitalized geriatric patients with symptomatic UTI in Sweden reported mainly GI AEs in both groups [33]. A multicenter RCT of pivmecillinam versus norfloxacin in women with symptoms of acute uUTI in Europe and Canada reported similar numbers of AEs and severe adverse drug reactions in both groups but higher rates of vaginal candidiasis and discontinuation due to AEs with norfloxacin [34].

In an active-controlled study versus cephalexin, 1 of 219 pivmecillinam-treated patients experienced a severe AE (nausea, lasting 2 days), whereas a severe AE of moniliasis, lasting 13 days, was reported in the cephalexin arm [38]. There were some indications of safety signals favoring pivmecillinam over other antibiotics. For example, a prospective randomized trial in patients with serious gram-negative infections in the United States reported AEs in 35% of patients treated with aminoglycoside versus 21% with mecillinam [35]. Notably, 15% (6/40) treated with aminoglycoside and 0% (0/38) treated with mecillinam had increased serum carnitine [35]. A Finnish trial comparing 3- and 10-day courses of both pivmecillinam and sulfadiazine/trimethoprim also revealed a difference in AE rate in favor of pivmecillinam at the longer course, including 3 AEs that led to discontinuation of sulfadiazine/trimethoprim [36].

### Comparison of Different Mecillinam/Pivmecillinam Dosage Regimens/Durations of Treatment

Ten studies compared different mecillinam/pivmecillinam dosage regimens or durations (Table 2) [29, 31, 39–46].

**Table 2.** Studies Comparing Different Mecillinam/Pivmecillinam Dosage Regimens or Durations of Treatment

Study	Design and Study Population	Treatment Groups^a^	Adverse Reactions, n (%)					Discontinued Due to AEs, n (%)
			Overall	GI Tract Related	Skin Related	Genitourinary	Other	
**Comparison of Pivmecillinam Duration**								
Jansåker et al, 2019 [29]	RCT; Denmark Non-pregnant women with dysuria, frequency, and/or urgency Age range 18–70 y	PIV 400 mg tid for 3 d (n = 156)	66 (42.3)	Diarrhea, nausea, vomiting, stomach ache, stomach discomfort: 44 (28.2)	Rashes, urticaria, pruritus, angioneurotic oedema: 2 (1.3)	Symptoms of vaginal/oral candidiasis: 9 (5.8)	Tiredness, vertigo/dizziness, headache: 27 (17.3) Depressive-like symptoms: 1 (0.6) Palpitations: 2 (1.3) Hot flush/sweating: 1 (0.6) Nosebleed: 1 (0.6) Shaking hands: 1 (0.6) Malaise: 1 (0.6)	NR
		PIV 400 mg tid for 5 d (n = 150)	51 (34.0)	Diarrhea, nausea, vomiting, stomach ache, stomach discomfort: 28 (18.7)	Rashes, urticaria, pruritus, angioneurotic oedema: 5 (3.3)	Symptoms of vaginal/oral candidiasis: 9 (6.0) Vaginal spotting: 1 (0.7)	Tiredness, vertigo/dizziness, headache: 25 (16.7) Depressive-like symptoms: 2 (1.3) Sleep problems: 1 (0.7) Muscle pain: 2 (1.3) Hot flush/sweating: 3 (2.0) Malaise: 1 (0.7)	NR
Marsh and Menday, 1980 [39]	RCT; UK Non-pregnant women with dysuria Age range 16–55 y	PIV for 3 d (n = 58)	10 (17.2)	Diarrhea: 5 (8.6) Nausea: 4 (6.9)	Skin rash: 2 (3.4)	NR	Headache: 2 (3.4) Tremor: 1 (1.7) Giddiness: 1 (1.7) Swollen ankles: 1 (1.7) Depression: 1 (1.7)	3 (2.3): diarrhea, nausea, tremor and giddiness; rash; nausea and vomiting
		PIV for 7 d (n = 67)	17 (25.4)	Diarrhea: 5 (7.5) Nausea: 6 (9.0) Vomiting: 1 (1.5)	Skin rash: 2 (3.0)	NR	Anorexia: 1 (1.5) Weakness: 1 (1.5) Giddiness: 1 (1.5) Lethargy: 1 (1.5)	
Sutlieff, 1982 [40]	RCT; UK Men and non-pregnant women with symptoms of dysuria Age range 18–55 y	PIV 400 mg initially then 200 mg tid (total 3 d; n = 44)	3 (6.8)	Nausea: 1 (2.3) Abdominal discomfort: 1 (2.3)	NR	NR	Dizziness: 1 (2.3) Headache: 1 (2.3) Drowsiness: 1 (2.3) Paresthesia: 1 (2.3)	NR
		PIV 200 mg bid for 5 d (n = 54)	9 (16.7)	Diarrhea: 2 (3.7) Vomiting: 1 (1.9) Nausea: 2 (3.7) Abdominal discomfort: 1 (1.9)	NR	Increased vaginal discharge: 2 (3.7)	Dizziness: 1 (1.9) Sore throat: 1 (1.9) Watering eyes: 1 (1.9)	NR
**Comparison of Pivmecillinam Dose**								
Skinner et al, 1984 [41]	Clinical trial; UK Non-pregnant female patients with acute UTI Age range 15–55 y	PIV 200 mg bid for 5 d (n = 185)	7 (3.8)	Nausea: 3 (1.6)	Rash: 1 (0.5)	Vaginal thrush: 1 (0.5)	Headache: 2 (1.1) Sore throat: 1 (0.5)	2 (1.1): nausea
		PIV 400 mg bid for 5 d (n = 111)	11 (9.9)	Nausea: 6 (5.4) Indigestion: 2 (1.8) Abdominal pains: 1 (0.9)	NR	Vaginal thrush: 1 (0.9)	Headache: 2 (1.8)	2 (1.8): nausea
Pettersson et al, 1984 [42]	RCT; country NR Acute UTI Age NR	PIV 200 mg tid for 7 d (n = 27)	3 (11.1)	GI: 2 (7.4)	NR	NR	Dysuria: 1 (3.7)	1 (3.7): persisting dysuria
		PIV 400 mg tid for 7 d (n = 31)	9 (29.0)	Nausea: 1 (3.2) Mild/moderate GI trouble and/or itching: 8 (25.8)	NR	NR	Headache: 1 (3.2) Dysuria: 1 (3.2)	1 (3.2): persisting dysuria, nausea, and headache
Mortimer et al, 1989 [43]	Retrospective analysis of AE reporting uUTI Age range 15–77 y (n = 31)	PIV 600 mg/d (n = 18)	18^b^	Esophageal ulceration: 15 Esophagitis: 3	NR	NR	NR	NR
		PIV 400 mg/d (n = 1)	1^b^	Esophagitis: 1	NR	NR	NR	NR
		PIV 200 mg/d (n = 1)	1^b^	Esophageal ulceration: 1	NR	NR	NR	NR
		PIV dose NR (n = 11)	11^b^	Esophageal ulceration: 11	NR	NR	NR	NR
Bresky, 1977 [31] ^c^	Prospective cohort; Sweden Patients with symptoms of acute UTI Mostly aged 15–30 y	PIV 400 mg tid for 10 d	23 (19)	Upper GI AEs (nausea, vomiting, anorexia, epigastric pain)	Typical exanthema: 1 Aggravation of pre-existing eczema: 1 Suspected mononucleosis with itching: 1 Mild transient skin reaction to face: 1	NR	NR	7
		PIV 200 mg tid for 10 d (n = 115)	13 (11)	Upper GI AEs: Heartburn/esophagitis: 2 Nausea/vomiting: 1	Tongue irritation:1 Conjunctivitis:1	…	…	5 (4.3): heartburn/esophagitis, nausea/vomiting, tongue irritation, conjunctivitis
**Comparison of Pivmecillinam Duration and Dose**								
Ferry et al, 2007 [44]	Prospective, double-blind RCT; Sweden Patients with symptoms of lower UTI Age range 18–89 y	Placebo (n = 227^d^)	(12)	GI: (4) Pyelonephritis: 1 (0.4)	NR	NR	NR	…
		PIV 200 mg tid for 7 d (n = 217^d^)	(17)	GI: (5–8) Pyelonephritis: 1 (0.2)	NR	NR	NR	7 (1.0)
		PIV 200 mg bid for 7 d (n = 220^d^)	(12)		NR	NR	NR	
		PIV 400 mg bid for 3 d (n = 220^d^)	(14)		NR	NR	NR	
		Control (n = 425)	NR	NR	NR	NR	NR	NR
Menday, 2002 [45]	Analysis of 2 RCTs; Study 1: Sweden; Study 2: Europe and Canada Women with symptomatic acute uncomplicated lower UTI Study 1: age ≥18 y; Study 2: age 18–65 y	Study 1: placebo (n = 289)	6 (2.1)	NR	NR	Vaginal candidiasis: 6 (2.1)	NR	NR
		Study 1: PIV 200 mg tid for 7 d (n = 282)	13 (4.6)	NR	NR	Vaginal candidiasis: 13 (4.6)	NR	NR
		Study 1: PIV 200 mg bid for 7 d (n = 294)	8 (2.7)	NR	NR	Vaginal candidiasis: 7 (2.4)	NR	NR
		Study 1: PIV 400 mg bid for 3 d (n = 287)	7 (2.4)	NR	NR	Vaginal candidiasis: 6 (2.1)	NR	NR
		Study 2: PIV 400 mg bid for 3 d (n = 479)	12 (2.5)	NR	NR	Vaginal candidiasis: 7 (1.5)	NR	NR
Pitkäjärvi et al, 1990 [46]	Open-label randomized study; Finland Patients with clinical signs of acute lower UTI Age range 16–65 y	PIV 400 mg tid for 3 d (n = 174)	17 (9.8)	Abdominal pain, nausea, diarrhea: 8 (4.6) Dryness of mouth: 2 (1.1)	Exanthema: 3 (1.7)	NR	Fatigue: 6 (3.4) Headache: 2 (1.1)	0
		PIV 200 mg tid for 7 d (n = 171)	18 (10.5)	Abdominal pain, nausea, diarrhea: 6 (3.5)	Exanthema: 2 (1.2) Genital itching: 2 (1.2)	Vaginitis/colpitis: 4 (2.3)	Fatigue: 3 (1.8) Headache: 1 (0.6) Insomnia: 2 (1.2)	0

Abbreviations: AE, adverse event; bid, twice daily; d, days; GI, gastrointestinal; h, hours; NR, not reported; PIV, pivmecillinam; RCT, randomized controlled trial; tid, 3 times daily; UTI, urinary tract infection; uUTI, uncomplicated urinary tract infection; y, years.

^a^n represents size of safety population.

^b^Note that this study preselected for cases of esophageal injury.

^c^Some n numbers and percentage data are missing from this entry as size of the safety population is unclear. Note that other details from this study are included in Table 1.

^d^n represents evaluated population (patients with significant bacteriuria).

A Danish RCT comparing 3- and 5-day courses of pivmecillinam 400 mg 3 times daily (tid) in women with uUTI reported a non-significant trend for a higher rate of GI AEs in the 3- versus 5-day arm (44/156 [28%] vs 28/150 [19%], respectively; *P* = .059), although symptoms suggestive of pyelonephritis developed in 2% of patients in each group [29]. Two UK RCTs assessed safety of pivmecillinam regarding treatment duration in patients with UTI; one compared a 3- versus 7-day course [39] and the other a 3- versus 5-day course [40]. In both studies, the AE rate was lower with the shorter course (10/58 [17%] vs 17/67 [25%] and 3/44 [6.8%] vs 9/54 [17%], respectively), but most AEs were mild with few or none leading to discontinuation.

Two studies assessed the effect of pivmecillinam dose on safety outcomes in acute UTI, comparing 200 mg and 400 mg twice daily (bid) over 5 days [41] or 200 mg and 400 mg tid over 7 days [42]. Fewer AEs were reported with the lower doses; most were mild or moderate and rarely led to discontinuation [41, 42]. These results corroborate data from a 1977 trial of pivmecillinam 400 mg tid versus amoxicillin, after which pivmecillinam 200 mg tid was studied in the same patient population (n = 115) and compared retrospectively with the full-dose group; AEs were reported in 11% and 19% patients, respectively [31]. Mortimer and colleagues (1989) examined 31 cases of esophageal complications related to different pivmecillinam doses for UTI, reported between 1978 and 1987 to the Swedish Adverse Drug Reactions Advisory Committee; the incidence of reported esophageal injuries was rare, at 2.0 cases per million defined daily doses [43]. Three further studies analyzed the impact of dosage and duration simultaneously [44–46]. A 7-day course of pivmecillinam 200 mg bid/tid was similarly tolerated to a 3-day course of pivmecillinam 400 mg bid/tid [44, 46]. Menday (2002) reported results of 2 randomized, double-blind clinical trials focusing on rates of vaginal candidiasis, which were similar across regimens (highest with 200 mg tid for 7 days) [45].

A systematic review and meta-analysis to explore optimal combinations of dosage, frequency, and duration of pivmecillinam in uncomplicated lower UTI used data from some studies identified in the current review [47]. Patients receiving a higher total pivmecillinam dose (2900–16 800 mg) were 40% (*P* = .062) and 44% (*P* = .293) more likely to report AEs than those treated with moderate (1900–2800 mg) or low (600–1800 mg) total doses, respectively. A moderate pivmecillinam dose given for 3 days was associated with 59% fewer AEs than patients treated with the same dose for 5 days (not statistically significant).

### Single-Arm Studies of Mecillinam/Pivmecillinam Monotherapy

Across the 7 single-arm studies of mecillinam/pivmecillinam monotherapy analyzed in detail (Table 3) [48–54], the most common events (incidence generally <5%) included nausea, diarrhea, vomiting, headache, backache, and rash [48–54]. One study reported serious AEs [54]. This was an open-label examination of pivmecillinam 400 mg 4 times daily (qid) for 7 days in older men and women (median age of 67 years) with comorbidities including diabetes, cancer, and indwelling catheter. Four SAEs were reported: 1 case of *Clostridioides difficile* infection (developed 2 weeks after cessation of pivmecillinam and subsequent cefotaxime therapy), and hospital readmissions due to cholecystitis, fever/worsened general condition, and spondylodiscitis. Two events caused pivmecillinam discontinuation (skin eruption and nausea/vomiting; non-serious) [54].

**Table 3.** Single-Arm Studies of Mecillinam/Pivmecillinam Monotherapy

Study	Design and Study Population	Treatment Groups^a^	Adverse Reactions, n (%)					Discontinued Due to AEs, n (%)
			Overall	GI Tract Related	Skin Related	Genitourinary	Other	
Cox, 1983 [48]	Non-comparative open-label study; country NR Men and women with UTI Age range 18–88 y	MEC 60 mg/kg per day for 7.8 mean therapy days (n = 48)	2 (4.2)^b^	NR	Pruritus: 1 (2.1) Skin rash: 1 (2.1)	NR	**Laboratory abnormalities:** Eosinophilia: 1 (2.1) Increase in serum creatinine level: 1 (2.1) Transient increases in SGOT and/or alkaline phosphatase: 2 (4.2)	6 (12.5): 2 AEs, 4 laboratory abnormalities
Place, 1981 [49]	Clinical trial; UK Patients with symptoms of frequency and dysuria or UTI Age range 18–55 y	PIV 200 mg tid for 5 d (n = 121)	8 (6.6)	Nausea: 4 (3.3) Diarrhea: 2 (1.7) Vomiting: 1 (0.8)	NR	NR	Dizziness: 2 (1.7) Dry mouth: 1 (0.8) Drowsiness: 1 (0.8) Lethargy: 1 (0.8)	1 (after 2 d): complaining of nausea and vomiting
Murison and Sweetenham, 1982 [50]	Clinical trial; UK Males and non-pregnant females with symptoms of dysuria Age range 18–55 y	PIV 200 mg tid for 5 d (n = 65)	4 (6.2)	Nausea: 2 (3.1) Loss of appetite: 1 (1.5)	NR	NR	Headache: 1 (1.5) Backache: 1 (1.5) Sweating: 1 (1.5) Muzzy head: 1 (1.5)	0
Donald and Rimmer, 1980 [51]	Clinical trial; UK Non-pregnant women with acute cystitis Age range 16–55 y	PIV initial dose of 400 mg (two tablets), followed by 200 mg tid for 3 d (n = 191)	16 (8.4)	Constipation: 1 (0.5) Diarrhea: 2 (1.0) Nausea: 10 (5.2) Vomiting: 3 (1.6) Flatulence: 1 (0.5) Abdominal distension: 1 (0.5)	Rash: 1 (0.5)	NR	Headache: 2 (1.0) Backache: 1 (0.5) Giddiness: 1 (0.5)	2 (1.0): 1 urticarial rash, 1 nausea and vomiting
Bresky and Lincoln, 1982 [52]	Prospective cohort; Sweden Males/females with chronic recurrent UTI Age range 18–71 y	PIV 200 mg tid for 11–17 wk (n = 30)	7 (23.3)	Esophageal pain: 1 (3.3) Vomiting: 2 (6.7) Gastric pain: 1 (3.3) Nausea: 3 (10.0)	Mild irritation of the skin in the axillae: 1 (3.3)	NR	Unusual sensation in the body: 3 (10.0), of which 2 cases associated with salt craving	3 (10.0) due to GI side effects: esophageal pain and nausea: 1 (3.3); vomiting and gastric pain: 1 (3.3); nausea and vomiting: 1 (3.3)
Diep et al, 1993 [53]	Analysis after long-term treatment; Norway Patients with chronic UTI, recurrent sinusitis, and chronic prostatitis 4 adults, 1 child	PIV 200 mg bid for 6 mo to 2 y (n = 4) or 500 mg tid for 6 mo (n = 1)	5 (study of patients with carnitine deficiency)	NR	NR	NR	NR	NR
Hansen et al, 2022 [54]	Prospective, single-arm uncontrolled study; Norway Men or women aged ≥18 y, hospitalized, with *E. coli* bacteremia due to febrile UTI Age range 34–87 y, 56% women	PIV 400 mg qid for 7 d (n = 53)	66 events	Abdominal pain: 6 events Diarrhea: 9 events Nausea/vomiting: 12 events Stomatitis: 1 event Esophagitis: 1 event **SAEs:** *Clostridioides difficile* infection: 1 event	Skin eruption/exanthema: 5 events	…	Headache: 8 events Dizziness: 10 events Other: 10 events **SAEs:** Readmitted to hospital due to cholecystitis: 1 event Readmitted to hospital due to fever/worsened general condition: 1 event Readmitted to hospital due to spondylodiscitis: 1 event	2 events: skin eruption, nausea/vomiting

Abbreviations: AE, adverse event; bid, twice daily; d, days; *E. coli*, *Escherichia coli*; GI, gastrointestinal; MEC, mecillinam; mo, months; NR, not reported; PIV, pivmecillinam; qid, 4 times daily; SAE, serious adverse event; SGOT, serum glutamic-oxaloacetic transaminase; tid, 3 times daily; UTI, urinary tract infection; wk, weeks; y, years.

^a^n represents size of safety population.

^b^Excluding laboratory test abnormalities, which were counted separately.

### Use of Mecillinam/Pivmecillinam in Pregnancy

Eleven studies analyzed safety outcomes of pivmecillinam and other antibiotics for bacteriuria during pregnancy (Table 4) [55–65]. These included 8 dedicated cohort or registry-based trials in Denmark [58–65], and 3 UK clinical trials of pivmecillinam including pregnant women [55–57].

**Table 4.** Studies Reporting Use of Mecillinam/Pivmecillinam During Pregnancy

A. Clinical Trials								
Study	Design and Study Population	Treatment Groups^a^	Adverse Reactions, n (%)					Discontinued Due to AEs, n (%)
			Overall	GI Tract Related	Skin Related	Genitourinary	Other	
Sanderson and Menday, 1984 [55]	Clinical trial; UK Women with bacteriuria in pregnancy (treated from between 10 and 28 wk of pregnancy) Age range 17–44 y	PIV 200 mg tid for 7 d (n = 44)	2 (4.5)	Diarrhea: 1 (2.3) Nausea and vomiting: 1 (2.3)	NR	NR	NR	2 (4.5): diarrhea: 1; nausea and vomiting: 1
		PIV 100 mg given prophylactically on alternate days (n = 12)	2 (16.7)	Nausea: 1 (8.3) Vomiting: 1 (8.3)	NR	NR	NR	2 (16.7): nausea: 1 (8.3); vomiting: 1 (8.3)
Brumfitt et al, 1979 [56]	Clinical trial; UK Non-pregnant individuals (n = 48) and pregnant women (n = 50) treated for bacteriuria Mean age across treatment groups 26.1–35.0 y	PIV 400 mg q6h for 7 d (n = 49)	16 (32.7)	10 (20.4) not specified	NR	Vaginal irritation: 3 (6.1)	Other: 4 (8.2) not specified	4 (8.2) not specified
		Cephradine 500 mg q6h for 7 d (n = 49)	25 (51.0)	11 (22.4) not specified	NR	Vaginal irritation: 15 (30.6)	Other: 5 (10.2) not specified	3 (6.1) not specified
Bint et al, 1979 [57]	Clinical trial; UK Women with bacteriuria of pregnancy	PIV 400 mg (capsules) qid for 7 d (n = 37)	21 (56.8)	Vomiting: 13 (35.1) Anorexia and/or nausea: 5 (13.5) Diarrhea: 1 (2.7) Indigestion: 1 (2.7) Epigastric fullness: 1 (2.7)	NR	NR	Headache: 2 (5.4) Dizzy and lightheaded: 1 (8.1)	8 (21.6) not specified
		PIV 400 mg (tablets) qid for 7 d (n = 12)	7 (58.3)	Anorexia and/or nausea: 3 (25.0) Vomiting: 1 (8.3)	NR	NR	Headache: 2 (16.7) Dizzy and lightheaded: 1 (8.3)	1 (8.3) not specified
		Ampicillin 500 mg qid for 7 d (n = 48)	11 (22.9)	Anorexia and/or nausea: 5 (10.4) Vomiting: 3 (6.3) Diarrhea: 2 (4.2)	Pruritus: 1 (2.1)	NR	Felt unwell: 1 (2.1)	1 (2.1) not specified
		PIV 200 mg tid for 7 d (low-dose follow-up; n = 20)	5 (25.0)	Vomiting: 1 (5.0) Anorexia and/or nausea: 4 (20.0)	NR	NR	Others: 2 (10.0)	1 (5.0) not specified

B. Dedicated Cohort or Registry-Based Trials				
Study	Design and Study Population	Analysis Groups	Treatments	Safety Outcomes
Mølgaard-Nielsen and Hviid, 2012 [58]	Registry; Denmark Singleton live births	First trimester (cleft lip with or without cleft palate): n = 18 First trimester (cleft palate alone): n = 13 Second month (cleft lip with or without cleft palate): n = 6 Third month (cleft palate alone): n = 9	PIV^b^	**Risk of isolated orofacial clefts, adjusted POR (95% CI):** First trimester (cleft lip with or without cleft palate): NR First trimester (cleft palate alone): NR Second month (cleft lip with or without cleft palate): NR Third month (cleft palate alone): 2.34 (1.20–4.54)
Larsen et al, 2001 [59]	Population-based follow-up study; Denmark	Pregnant women who redeemed prescriptions for pivmecillinam: n = 414 Reference group (pregnant women for whom no drugs were prescribed during pregnancy): n = 7472	PIV	**Pivmecillinam prescriptions during first trimester:** Number of congenital abnormalities: 2 Number of children with low birth weight^c^: 4 (1) Number of preterm deliveries: 5 Number of stillborn babies (before birth): 1 Number of stillborn babies (during birth): 1 **Pivmecillinam prescribed at any time during pregnancy:** Number of congenital abnormalities: 12 (adjusted OR 0.46; 95% CI: .11–1.86) Number of children with low birth weight^c^: 17 (5) (adjusted OR 0.57; 95% CI: .23–1.41) Number of preterm deliveries: 24 (adjusted OR 0.91; 95% CI: .60–1.40) Number of stillborn babies (before birth): 1 Number of stillborn babies (during birth): 1 **Reference group:** Number of congenital abnormalities: 274 Number of children with low birth weight^c^: 402 (141) Number of preterm deliveries: 480 Number of stillborn babies (before birth): 20 Number of stillborn babies (during birth): 7
Skriver et al, 2004 [60]	Observational study; Denmark	Prescriptions taken up during entire pregnancy: n = 2031 Prescriptions taken up within first trimester: n = 559 Prescriptions taken up 28 d before delivery: n = 371 No prescriptions for pivmecillinam taken up during pregnancy: n = 61 628	PIV	**Risk of adverse birth and neonatal outcomes, adjusted OR (95% CI); exposure 28 d before delivery versus unexposed pregnancies** Birth defects following PIV exposure during first trimester: 0.83 (0.53–1.32) Birth defects without chromosomal abnormalities: 0.83 (0.53–1.32) Preterm delivery: 0.96 (0.79–1.18) Low birth weight following PIV exposure at any time during pregnancy: 0.79 (0.52–1.20) Stillbirth: 1.19 (0.30–4.80) Low Apgar score: 1.17 (0.37–3.66) Hypoglycemia: 1.03 (0.53–2.00) Respiratory distress syndrome: 0.79 (0.38–1.68)
Damkier et al, 2019 [61]	Cohort study; Denmark Singleton pregnancies resulting in a live birth of an infant without chromosomal abnormalities	Overall population: Exposed to study antibiotic: n = 82 318 Exposed to reference penicillin: n = 48 765 Unexposed population: n = 801 648	PIV, sulfamethizole, and azithromycin	Exposed to study antibiotic: inferential analysis did not identify any association between exposure and any of the 3 outcomes (major congenital malformations, cardiac malformation, and any malformation), versus primary control cohort (penicillin group) in the fully adjusted model Any malformations: 2291 Major malformations: 1286 Cardiac malformations: 380 **Compared with unexposed pregnancies:** *Pivmecillinam* Risks for major malformations: OR 1.13; 95% CI: 1.06–1.19 Risk for cardiac malformations: OR 1.15; 95% CI: 1.04–1.28 *Sulfamethizole* Risks for major malformations: OR, 1.15; 95% CI: 1.07–1.24 Risk for cardiac malformations: OR, 1.22; 95% CI: 1.07–1.39 *Azithromycin* Risks for major malformations: OR, 1.19; 95% CI: 1.03–1.38 Risk for cardiac malformations: OR, 1.29; 95% CI: .99–1.67
Miller et al, 2012 [62]	Registry; Denmark Children diagnosed with epilepsy	Mothers unexposed, at any time during pregnancy, to any cystitis antibiotic: n = 378 809 Mothers exposed, at any time during pregnancy, to any cystitis antibiotic: n = 68 820 Mothers exposed, at any time during pregnancy, to pivmecillinam: n = 34 609 One redeemed prescription (pivmecillinam, sulfamethizole, and nitrofurantoin): n = 36 072 One redeemed prescription (pivmecillinam): n = 15 975 >1 redeemed prescription (pivmecillinam, sulfamethizole, and nitrofurantoin): n = 32 748 >1 redeemed prescription (pivmecillinam): n = 18 634	PIV, sulfamethizole, nitrofurantoin	**Risk of childhood epilepsy, n (%)** Mothers unexposed, at any time during pregnancy, to any cystitis antibiotic: 2348 (82) Mothers exposed, at any time during pregnancy, to any cystitis antibiotic: 500 (18) Mothers exposed, at any time during pregnancy, to pivmecillinam: 233 (8) One redeemed prescription (pivmecillinam, sulfamethizole, and nitrofurantoin): 232 (8) One redeemed prescription (pivmecillinam): 96 (3) >1 redeemed prescriptions (pivmecillinam, sulfamethizole, and nitrofurantoin): 268 (9) >1 redeemed prescriptions (pivmecillinam): 137 (5) **Risk of childhood epilepsy versus unexposed pregnancies, adjusted HR (95% CI)** Mothers exposed, at any time during pregnancy, to any cystitis antibiotic: 1.2 (1.1–1.3) Mothers exposed, at any time during pregnancy, to pivmecillinam: 1.2 (1.0–1.4) One redeemed prescription (pivmecillinam, sulfamethizole, and nitrofurantoin): 1.1 (0.9–1.2) One redeemed prescription (pivmecillinam): 1.1 (0.9–1.3) >1 redeemed prescription (pivmecillinam, sulfamethizole, and nitrofurantoin): 1.3 (1.2–1.5) >1 redeemed prescription (pivmecillinam): 1.3 (1.1–1.5)
Miller et al, 2013 [63]	Registry (population-based cohort); Denmark Children with a diagnosis of febrile seizures and children born to women who took at least 1 type of antibiotic during pregnancy (exposed) and children of women who did not take any antibiotics during pregnancy (unexposed)	**Exposed to:** Pivmecillinam: n = 34 596 Penicillin V: n = 79 063 Sulfamethizole: n = 37 648 Erythromycin: n = 15 886 Nitrofurantoin: n = 6926 **Unexposed:** N = 378 639	PIV, penicillin V, sulfamethizole, erythromycin, nitrofurantoin	**1. Risk of febrile seizures in the children, according to antibiotic exposure, n; adjusted HR (95% CI):** Unexposed: 14 550; reference value Pivmecillinam: 1508; 1.12 (1.06–1.18) Penicillin V: 3242; 1.06 (1.02–1.10) Sulfamethizole: 1649; 1.12 (1.06–1.18) Erythromycin: 638; 1.03 (0.95–1.11) Nitrofurantoin: 312; 1.16 (1.04–1.29) Any systemic antibiotic: 1.08 (1.05–1.11) **2. Risk of febrile seizures for children born at term with a birth weight >2500 g, no congenital malformations, and an Apgar score at 5 min of >10, n; adjusted HR (95% CI):** Unexposed: 10 942; reference value Pivmecillinam: 1142; 1.13 (1.06–1.20) Penicillin V: 2472; 1.06 (1.02–1.11) Sulfamethizole: 1219; 1.11 (1.05–1.18) Erythromycin: 470; 1.00 (0.92–1.10) Nitrofurantoin: 235; 1.16 (1.02–1.32) Any systemic antibiotic: 1.08 (1.04–1.11) **3. Risk of febrile seizures in the children, by number of redeemed prescriptions during pregnancy, one redemption, n; adjusted HR (95% CI):** Unexposed: 14 550; reference value Pivmecillinam: 694; 1.12 (1.04–1.21) Penicillin V: 1826; 1.03 (0.98–1.08) Sulfamethizole: 758; 1.08 (1.00–1.16) Erythromycin: 274; 1.06 (0.94–1.19) Nitrofurantoin: 94; 1.17 (0.95–1.43) Any systemic antibiotic: 1.06 (1.03–1.09) **4. Risk of febrile seizures in the children, by number of redeemed prescriptions during pregnancy, > 1 redemption, n; adjusted HR (95% CI):** Unexposed: 14 550; reference value Pivmecillinam: 814; 1.12 (1.04–1.20) Penicillin V: 1416; 1.10 (1.04–1.16) Sulfamethizole: 891; 1.16 (1.08–1.24) Erythromycin: 364; 1.00 (0.90–1.11) Nitrofurantoin: 218; 1.16 (1.01–1.32) Any systemic antibiotic: 1.11 (1.06–1.15)
Nørgaard et al, 2008 [64]	Case-control study; Denmark Cases – defined as women who, during the study period, had a first-time recorded miscarriage and no previously recorded birth Control – women with a first live birth during the study period and no previous recorded miscarriage in the Hospital Discharge Registry	**Cases (n = 1599)** Pivmecillinam exposure: n = 17 Sulfamethizole exposure: n = 37 Penicillin V exposure: n = 47 NSAID exposure: n = 45 **Controls (n = 15 990)** Pivmecillinam exposure: n = 145 Sulfamethizole exposure: n = 394 Penicillin V exposure: n = 463 NSAID exposure: n = 297	PIV, sulfamethizole, penicillin V, NSAIDs	**Risk of miscarriage, adjusted OR (95% CI):** *Pivmecillinam* Within 1 wk before hospitalization: 2.03 (0.77–5.33) Within 2–3 wk before hospitalization: 1.10 (0.44–2.78) Within 4–7 wk before hospitalization: 0.75 (0.27–2.10) Within 8–12 wk before hospitalization: 1.27 (0.38–4.22) *Sulfamethizole* Within 1 wk before hospitalization: 1.53 (0.76–3.09) Within 2–3 wk before hospitalization: 0.92 (0.47–1.82) Within 4–7 wk before hospitalization: 1.04 (0.61–1.77) Within 8–12 wk before hospitalization: 0.52 (0.19–1.43) *Penicillin V* Exposure to penicillin V in the week before the miscarriage was not associated with an increased risk of miscarriage (OR, 1.03; 95% CI: .41–2.61) Penicillin V (controls: reference) *NSAIDs* When including use of NSAIDs, the risk estimates did not change substantially in any of the analyses (data not shown)
Nørgaard et al, 2012 [65]	Registry; Denmark Singletons born	Pivmecillinam: n = 17 756 Penicillin V: n = 27 150 Other penicillins: n = 12 259 Sulfonamides/trimethoprim: n = 12 748 Macrolides: n = 5847 Unexposed: n = 131 307	PIV, penicillin V, sulfonamides/trimethoprim, macrolide, other penicillin	**Pivmecillinam:** Risk of childhood epilepsy, n = 106 Adjusted IRR (95% CI): 1.55 (1.25–1.93) **Penicillin:** *Penicillin V* Risk of childhood epilepsy, n = 177 Adjusted IRR (95% CI): 1.56 (1.30–1.87) *Other penicillins* Risk of childhood epilepsy, n = 79 Adjusted IRR (95% CI): 1.46 (1.15–1.87) **Sulfonamides/trimethoprim:** Risk of childhood epilepsy, n = 83 Adjusted IRR (95% CI): 1.42 (1.12–1.82) **Macrolides:** Risk of childhood epilepsy, n = 46 Adjusted IRR (95% CI): 1.61 (1.15–2.25) **Unexposed:** Risk of childhood epilepsy, n = 587; reference value

Abbreviations: AE, adverse event; CI, confidence interval; d, days; GI, gastrointestinal; HR, hazard ratio; IRR, incidence rate ratio; NR, not reported; NSAID, non-steroidal anti-inflammatory drug; OR, odds ratio; POR, prevalence odds ratio; PIV, pivmecillinam; q6h, every 6 hours; qid, 4 times daily; tid, 3 times daily; wk, weeks; y, years.

^a^n represents size of safety population.

^b^Study reports data for other antibiotics also.

^c^Full-term deliveries are given in parentheses.

One UK trial compared pivmecillinam 400 mg (capsule/tablet formulation) qid for 7 days with ampicillin 500 mg qid for 7 days in 100 pregnant patients with bacteriuria [57]. There were significant differences in favor of ampicillin versus pivmecillinam for rates of 1 or more side effects (*P* < .01), vomiting (*P* < .01), and premature discontinuation (*P* < .02). Twenty patients subsequently received pivmecillinam at the lower dose of 200 mg tid for 7 days; incidence of side effects was markedly reduced and was similar to that previously recorded with ampicillin [57]. The other 2 clinical studies analyzed pivmecillinam as a treatment or prophylactic regimen [55] or compared pivmecillinam with cephradine [56] in pregnant women with bacteriuria (the latter study also included non-pregnant women). GI AEs were the most frequently reported AEs and rates of discontinuation due to AEs were low. In the comparative study, vaginal irritation occurred in 30.6% of cephradine-treated patients versus 6.1% in the pivmecillinam group [56].

The dedicated cohort or registry-based trials identified a number of risks associated with antibiotic use that included but were not specific to pivmecillinam. In one study, compared with antibiotic-unexposed pregnancies, an increased risk of cleft lip with or without cleft palate was reported with second month use of doxycycline/tetracycline (prevalence odds ratio [POR] 7.30; 95% confidence interval [CI]: 1.81–29.46) or sulfamethizole (POR 1.76; 95% CI: 1.10–2.81) [58]. An increased risk of cleft palate was seen for first-trimester use of trimethoprim (POR 14.29; 95% CI: 3.46–59.05) or use of pivmecillinam in the third month of pregnancy (POR 2.34; 95% CI: 1.20–4.54) [58]. Another study suggested that first-trimester exposure to 10 commonly prescribed antibiotics (including pivmecillinam) was not associated with an increased risk of congenital malformations versus a control group exposed to penicillins that are not considered to carry any risk of major congenital malformations [61]. A large population-based cohort study examined whether maternal use of antibiotics during pregnancy was associated with increased risk of childhood epilepsy, reporting an adjusted hazard ratio (95% CI) of 1.2 (1.0–1.4) for pivmecillinam, 1.2 (1.1–1.4) for sulfamethizole, and 1.1 (0.8–1.5) for nitrofurantoin [62]. Another study reported that, among individuals prenatally exposed to pivmecillinam, the adjusted incidence rate ratio (IRR) for childhood epilepsy was 1.55 (95% CI: 1.25–1.93). For the other antibiotic groups listed (penicillin V, other penicillins, sulfonamide/trimethoprim, and macrolides), adjusted IRRs ranged from 1.42 to 1.61 [65]. Miller and colleagues (2013) identified weak associations between use of certain antibiotics during pregnancy and febrile seizures in early childhood [63]. Two population-based cohort studies concluded that pivmecillinam was not associated with a significantly increased risk of adverse birth outcome [59, 60].

### Use of Mecillinam/Pivmecillinam in the Pediatric Population

Four studies reported use of mecillinam/pivmecillinam in pediatric patients with recurrent [66] or acute UTI [67], shigellosis [68], or acute pyelonephritis [69] (Table 5). Drug-related AEs were generally mild. In patients treated for UTI, GI- and skin-related AEs were observed as well as tiredness/irritability and fatigue [66, 67]. In the prospective, randomized, double-blind trial in 59 children with shigellosis, vomiting was more frequent for all days of treatment in patients treated with TMP-SMX than pivmecillinam, and mild elevation of aminotransferases occurred in 4/30 (13.3%) versus 5/29 (17.2%), respectively [68].

**Table 5.** Studies Reporting Use of Pivmecillinam in Pediatric Patients

Study	Design and Study Population	Treatment Groups^a^	Adverse Reactions, n (%)					Discontinued Due to AEs, n (%)
			Overall	GI Tract Related	Skin Related	Genitourinary	Other	
Carlsen et al, 1985 [66]	Open-label, randomized, crossover study; Denmark Children with a history of vesicoureteral reflux or with recurrent UTI Age range 1–13 y	PIV 100 mg qd for children aged <6 y and bid for children aged >6 y, for 10 d (n = 33)	5 (15.2)	Loose stools: 1 (3.0) Dyspepsia, stomach pain: 2 (6.1)	NR	NR	Anorexia: 1 (3.0) Tiredness: 2 (6.1)	0 (0)
		Nitrofurantoin 1.5 mg/kg, for 10 d (n = 32)	10 (31.3)	Loose stools: 1 (3.1) Obstipation: 1 (3.1) Dyspepsia, stomach pain: 3 (3.1) Difficulties swallowing, bad taste: 2 (6.3) Nausea, stomach pain 1 (3.1)	Urticarial rash: 1 (3.1)	NR	Anorexia: 4 (12.5) Tiredness: 1 (3.1) Peripheral paresthesia: 1 (3.1)	5 (15.6): urticaria 1 (3.1); nausea and stomach pain 1 (3.1); peripheral paresthesia 1 (3.1); dyspepsia 1 (3.1); stomach pain 1 (3.1)
Petersen, 1991 [67]	Prospective, open-label RCT; Denmark Children with UTI Mean age across treatment groups 7.5–7.9 y	PIV 20–40 mg/kg/24 h bid for 3 d (n = 117)	6 (5.1)	Vomiting and abdominal pain: 2 (1.7) Diarrhea: 1 (0.9)	Urticarial rash: 2 (1.7)	NR	Irritability and fatigue: 1 (0.9)	2 (1.7): vomiting and abdominal pain
		Sulfamethizole 40–80 mg/kg/24 h bid for 3 d (n = 121)	2 (1.7)	GI complaints of diarrhea, vomiting, and abdominal pain: 2 (1.7)	NR	NR	NR	1 (0.8): GI complaints
		Sulfamethizole 40–80 mg/kg/24 h bid for 10 d (n = 121)	0 (0)	NR	NR	NR	NR	NR
Prado et al, 1993 [68]	Prospective, double-blind RCT; US Children with shigellosis Age range 0.5–13 y	PIV for 5 d: Bottle: 5 mL (<20 kg) and 10 mL (>20 kg) Sachets: 40 mg/kg/day (n = 29)	NR	NR	NR	NR	Mild elevation of aminotransferases: 5 (17.2)	NR
		TMP-SMX for 5 d: Bottle: 5 mL (<20 kg) and 10 mL (>20 kg) Sachets: 40 mg/kg/day (n = 30)	NR	NR	NR	NR	Mild elevation of aminotransferases: 4 (13.3)	NR
Sehested et al, 2021 [69]	Prospective, multicenter study; Denmark Children with acute pyelonephritis Age range 0.5–16 y	PIV 20–40 mg/kg/d for 10 d (n = 74)	NR	(Reason for switching to IV antibiotics) Vomiting: 8	NR	NR	(Reason for switching to IV antibiotics) Positive blood culture and lack of clinical improvement: 4 Increasing plasma creatinine: 1	NR
		Amoxicillin-clavulanate: 40–50 mg amoxicillin/kg/d for 10 d (n = 344)	NR		NR	NR		NR

Abbreviations: AE, adverse event; bid, twice daily; d, days; GI, gastrointestinal; h, hours; IV, intravenous; NR, not reported; PIV, pivmecillinam; RCT, randomized controlled trial; TMP-SMX, trimethoprim-sulfamethoxazole; UTI, urinary tract infection; y, years.

^a^n represents size of safety population.

### Safety of Mecillinam/Pivmecillinam Used in Combination With Other Antibiotics

Safety outcome data were identified relating to use of mecillinam/pivmecillinam in combination with other beta-lactams (ampicillin, cephalothin, cefamandole, cefoxitin, cefuroxime, cephalosporin, or pivampicillin) [70–73], including 18 studies of the fixed-dose combination of pivmecillinam and pivampicillin [74–91] (Supplementary Table 6). AE profiles were generally as expected based on individual class effects. Two studies of long-term treatment with the fixed-dose combination demonstrated reductions in serum carnitine concentration to approximately 10%–15% of pretreatment/reference values [88, 89].

## DISCUSSION

Owing to the long history of clinical use of pivmecillinam in Europe and Canada, there is a substantial body of safety data available, largely in the UTI setting. It is impossible to distinguish between safety issues due to active moiety versus administration form, so this review considered data on both pivmecillinam and mecillinam. Studies typically reported doses of pivmecillinam hydrochloride, 200 mg of which is equivalent to 185 mg of pivmecillinam as approved for use in the United States [3].

Beta-lactam antibiotics are recognized for their favorable safety profile, with SAEs rarely reported [92]. Many studies reported either no or mild/very mild AEs during pivmecillinam treatment; in others, the most frequently reported AEs were GI in nature (particularly upper GI) including nausea, diarrhea, and vomiting; typically occurring in fewer than 5% of treated patients [43]. Esophageal injury, which is mainly associated with tetracyclines, has rarely been reported with pivmecillinam, and has not caused the long-term complications observed with other drugs [43]. Esophageal tolerability was also improved through reformulation of the tablet with a disintegrator [93]. Other AEs included skin reactions such as rash/exanthema, and events such as headache, lethargy/fatigue, and laboratory test abnormalities. Discontinuation of pivmecillinam due to AEs was uncommon (typically <2%, sometimes nil [30, 46, 50, 66]). Typical AE profiles and discontinuation rates in comparative studies were generally at least as good for pivmecillinam 200 mg as for other antibiotics. There was a non-significant trend for increased AE risk with the 400-mg compared with the 200-mg dose (bid or tid) [41, 42, 46]. The rate of AEs was similar with 400 mg tid for 3 days versus 200 mg tid for 7 days [46].

Several studies reported vaginal candidiasis cases following pivmecillinam treatment [34, 36, 41, 45, 46]. Increased risk of this microbiome-related AE has been demonstrated with use of broad- and narrow-spectrum beta-lactam antibiotics [19]. However, as pivmecillinam exerts minimal impact on the vaginal biome [94], it should be associated with a relatively low risk of vaginal candidiasis. Indeed, one study demonstrated a nearly three-fold lower rate of vaginal candidiasis with pivmecillinam versus norfloxacin [34].

Antibiotics used to treat UTI are subject to rare but notable safety events. SAEs have rarely been reported with pivmecillinam; however, carnitine depletion is an important consideration [3]. Treatment with pivalic acid–containing drugs, such as pivampicillin and pivmecillinam, can deplete carnitine stores as pivalic acid forms an ester with carnitine, pivaloylcarnitine, which is excreted in the urine [88, 89, 95]. No clinical effects of decreased carnitine have been associated with short-term pivmecillinam treatment; however, treatment is contraindicated in patients with primary or secondary carnitine deficiency resulting from inherited disorders of mitochondrial fatty acid oxidation and carnitine metabolism, and other inborn errors of metabolism [3]. Although pivmecillinam is not recommended for prolonged treatment, depleted carnitine levels apparently recover to normal without carnitine supplementation after cessation of long-term pivmecillinam treatment [53].

A number of studies reported no significantly increased risk in adverse birth outcome in pivmecillinam-treated pregnant women versus unexposed pregnancies [55, 59, 60]. The US Prescribing Information states that published observational studies during the first trimester do not indicate an increased risk of major birth defects. The few studies on pivmecillinam use during pregnancy that evaluated risk of miscarriage and other adverse maternal or fetal outcomes have had methodological limitations. No dose adjustment is recommended in pregnant women [3].

The US indication for pivmecillinam does not include pediatric patients [3]; however, it is approved for patients aged 6 years and over in Canada [6] and in adults and children (with weight-based dose reduction for children weighing <40 kg) in Europe [4, 5]. This review identified four studies in pediatric patients; the safety profile of pivmecillinam was generally similar to that established in adults [66–69].

Comparative interpretation of the reviewed data was limited by considerable heterogeneity between study designs and reporting. Few data were available to compare pivmecillinam with other first-line uUTI medications, such as nitrofurantoin or a 3-day course of TMP-SMX.

## CONCLUSIONS

This review of an extensive historical dataset establishes the benign safety and tolerability profile of pivmecillinam. Together with the evidence for efficacy of, and minimal resistance to, pivmecillinam in the treatment of uUTI, these data will help to inform treatment decisions following its recent US FDA approval in this indication and recommendations of pivmecillinam as a first-line treatment for uUTIs. The availability of pivmecillinam represents a long-awaited addition to the US physician's armamentarium for tackling this highly prevalent and burdensome condition.

## Supplementary Data

Supplementary materials are available at *Clinical Infectious Diseases* online. Consisting of data provided by the authors to benefit the reader, the posted materials are not copyedited and are the sole responsibility of the authors, so questions or comments should be addressed to the corresponding author.

## Supplementary Material

ciae621_Supplementary_Data

## Notes

***Author Contributions.*** Study conception and design: A. S. H. Data curation and formal analysis: A. S. H. Writing—original draft: All authors. Writing–review and editing: All authors.

***Acknowledgments.*** Medical writing support was provided by Becky Fox-Spencer, PhD, and Anna Palmer, PhD, both contracted by LiNK Health Group, LLC.

***Data sharing.*** Data in this systematic review were extracted from published studies.

***Financial support*.** This work was supported by UTILITY therapeutics, who funded the provision of medical writing assistance to the authors.
