# Supplementary material for: Safety and Tolerability of Pivmecillinam During More Than Four Decades of Clinical Experience: A Systematic Review
Source: Clin Infect Dis. 2025 Jan 21;80(2):280–99. doi: 10.1093/cid/ciae621 (PMC11848268; doi:10.1093/cid/ciae621)
Supplement: ciae621_Supplementary_Data [file ciae621_supplementary_data.docx]

SUPPLEMENTARY DATA

Safety and Tolerability of Pivmecillinam During More Than Four Decades of Clinical Experience: A Systematic Review

**Correspondence to:** Keith S. Kaye ([kk1116@rwjms.rutgers.edu](mailto:kk1116@rwjms.rutgers.edu))

**CONTENTS**

**Supplementary Table 1.** PICOS eligibility criteria **Supplementary Table 2**. PubMed search strategy and hits **Supplementary Table 3**. Embase search strategy and hits
**Supplementary Table 4.** Characteristics of included studies **Supplementary Table 5.** Studies not summarized in main tables

**Supplementary Table 6.** Studies of mecillinam/pivmecillinam used in combination (including fixed-dose combination of pivmecillinam and pivampicillin)

**Supplementary Table 1. PICOS Eligibility Criteria**

| **Domain** | **Inclusion Criteria** | **Exclusion Criteria** |
| --- | --- | --- |
| **Population** | Patients with urinary tract or any other infection who received pivmecillinam or mecillinam | - |
| **Interventions** | Pivmecillinam, mecillinam, amdinocillin | - |
| **Comparators** | No restrictions | - |
| **Outcomes** | Patient characteristics  Safety  Adverse events  Any adverse event  Severe adverse events  Serious adverse events  Side effects  Abdominal pain  Anaphylactic reactions  Carnitine deficiency  Carnitine transporter defect  *Clostridium difficile* colitis  Confusion  Diarrhea  Dizziness  Dyspepsia  Eosinophilia  Fatigue  Headache  Hepatic function abnormal  Methylmalonic aciduria  Mouth ulcer  Muscle aches  Nausea  Neutropenia  Esophageal ulceration  Esophagitis  Porphyria  Propionic acidemia  Pruritus  Pseudomembranous colitis  Rash (skin)  Thrombocytopenia  Urticaria  Vaginal candidiasis  Vaginitis  Vertigo  Vomiting  Vulvovaginal mycotic infection | Clinical efficacy outcomes only  Quality of life outcomes only  Cost and economic impact outcomes only |
| **Study design** | Randomized controlled trials  Systematic reviews and meta-analyses  Observational studies  Case reports/case series (with N≥10)  Letters (with data) | Animal, *in vitro* studies  Economic evaluations  Narrative reviews, letters, and comment articles  Notes, editorial, correspondence, opinion |
| **Language** | English | Non-English |
| **Time period** | Inception to October 24, 2023 | - |

Abbreviation: PICOS, population, intervention, comparator, outcome, and study type.

**Supplementary Table 2. PubMed Search Strategy and Hits**

| **Strategy** | **ID#** | **Query** | **Hits** |
| --- | --- | --- | --- |
| **PIV** | #1 | "Amdinocillin Pivoxil"[MeSH] OR "Amdinocillin Pivoxil" OR Pivamdinocillin OR Pivmecillinam* OR "Mecillinam Pivaloyl Ester" OR Selexid OR "Pivmecillinam Hydrochloride" OR "FL-1039" OR "FL 1039" OR FL1039 OR coactabs OR selexid OR ro109071 OR penomax OR mecillinam | 403 |
| **PIV and clinical trials** | #2 | #1 AND "clinical study" OR "clinical trial" OR "clinical studies" OR "clinical trials" OR "clinical data" OR "clinical evidence" OR "clinical evidences" | 121 |
| **PIV and RCTs** | #3 | #1 AND ("randomized controlled" OR "randomised controlled" OR "randomized-controlled" OR "randomised-controlled" OR RCT OR RCTs) | 88 |
| **PIV and (clinical trials or RCTs)** | #4 | #1 AND ("clinical study" OR "clinical trial" OR "clinical studies" OR "clinical trials" OR "clinical data" OR "clinical evidence" OR "clinical evidences" OR "randomized controlled" OR "randomised controlled" OR "randomized-controlled" OR "randomised-controlled" OR RCT OR RCTs) | 136 |
| **PIV and safety** | #5 | #1 AND ("Abdominal Pain"[MeSH] OR "Anaphylaxis"[MeSH] OR ("Anaphylaxis"[MeSH]) AND "Systemic carnitine deficiency" [Supplementary Concept] OR "Systemic carnitine deficiency" [Supplementary Concept] OR "Confusion"[MeSH] OR "Diarrhea"[MeSH] OR "Dizziness"[MeSH] OR "Dyspepsia"[MeSH] OR "Eosinophilia"[MeSH] OR "Fatigue"[MeSH] OR "Headache"[MeSH] OR "Liver Diseases"[MeSH] OR "Oral Ulcer"[MeSH] OR "Myalgia"[MeSH] OR "Nausea"[MeSH] OR "Neutropenia"[MeSH] OR "Esophagitis"[MeSH] OR "Porphyrias"[MeSH] OR "Propionic Acidemia"[MeSH] OR "Pruritus"[MeSH] OR "Enterocolitis, Pseudomembranous"[MeSH] OR "Exanthema"[MeSH] OR "Thrombocytopenia"[MeSH] OR "Urticaria"[MeSH] OR "Candidiasis, Vulvovaginal"[MeSH] OR "Vaginitis"[MeSH] OR "Valproic Acid"[MeSH] OR "Vertigo"[MeSH] OR "Vomiting"[MeSH] OR Safety OR "adverse events" OR "adverse-events" OR "adverse effects" OR "adverse-effects" OR AEs OR SAEs OR "Side effects" OR "side effects" OR "Abdominal pain" OR "Anaphylactic reactions" OR "Carnitine deficiency" OR "Carnitine depletion" OR "Carnitine transporter defect" OR "Clostridium difficile colitis" OR Confusion OR Diarrhea OR Dizziness OR Dyspepsia OR Eosinophilia OR Fatigue OR Headache OR "Hepatic function abnormal" OR "Methylmalonic aciduria" OR "Mouth ulcer" OR "Muscle aches" OR Nausea OR Neutropenia OR "Oesophageal ulceration" OR "Oesophageal ulcer" OR "Oesophagitis" OR "Porphyria" OR "Propionic acidaemia" OR Pruritus OR "Pseudomembranous colitis" OR "Rash skin" OR "Thrombocytopenia" OR Urticaria OR "Vaginal candidiasis" OR Vaginitis OR Valproate OR "Valproic acid" OR Vertigo OR Vomiting OR "Vulvovaginal mycotic infection" OR "Drug-Related Side Effects and Adverse Reactions"[MeSH] OR "Adverse Drug Reaction Reporting Systems"[MeSH]) | 165 |
| **Total PIV and (clinical trials or RCTs) + PIV and safety** | #4 + #5 |  | 301 |

Abbreviations: AE, adverse event; MeSH, Medical Subject Headings; PIV, pivmecillinam; RCT, randomized controlled trial; SAE, serious adverse event.

**Supplementary Table 3. Embase Search Strategy and Hits**

| **Strategy** | **ID#** | **Query** | **Hits** |
| --- | --- | --- | --- |
| **PIV** | #6 | 'pivmecillinam'/exp OR 'amdinocillin pivoxil' OR pivamdinocillin OR pivmecillinam* OR 'mecillinam pivaloyl ester' OR selexid OR 'pivmecillinam hydrochloride' OR 'fl 1039' OR 'fl 1039' OR coactabs OR ro109071 OR penomax OR 'mecillinam'/exp OR mecillinam | 2721 |
| **PIV and clinical trials** | #7 | #6 AND ('clinical study'/exp OR 'clinical study' OR 'clinical trial' OR 'clinical studies' OR 'clinical trials' OR' clinical data' OR 'clinical evidence' OR 'clinical evidences') | 958 |
| **PIV and RCTs** | #8 | #6 AND ('randomized controlled trial'/exp OR 'randomized controlled' OR 'randomised controlled' OR 'randomized-controlled' OR 'randomised-controlled' OR rct OR rcts) | 136 |
| **PIV and (clinical trials or RCTs)** | #9 | #6 AND ('clinical study'/exp OR 'clinical study' OR 'clinical trial' OR 'clinical studies' OR 'clinical trials' OR' clinical data' OR 'clinical evidence' OR 'clinical evidences' OR 'randomized controlled trial'/exp OR 'randomized controlled' OR 'randomised controlled' OR 'randomized-controlled' OR 'randomised-controlled' OR rct OR rcts) | 974 |
| **PIV and safety** | #10 | #6 AND ('adverse event'/exp OR 'adverse drug reaction'/exp OR 'drug safety'/exp OR 'side effect'/exp OR Safety OR 'adverse events' OR 'adverse-events' OR' adverse effects' OR 'adverse-effects' OR AEs OR SAEs OR 'side effects' OR 'side effects' OR 'abdominal pain'/exp OR 'anaphylactic reactions' OR 'carnitine deficiency'/exp OR 'carnitine depletion' OR 'carnitine transporter defect' OR 'clostridium difficile colitis' OR 'confusion' OR 'diarrhea' OR 'dizziness' OR 'dyspepsia' OR 'eosinophilia' OR 'fatigue' OR 'headache' OR 'hepatic function abnormal' OR 'methylmalonic aciduria' OR 'mouth ulcer' OR 'mouth ulceration' OR 'muscle aches' OR 'nausea' OR 'neutropenia' OR 'oesophageal ulceration' OR 'oesophageal ulcer' OR oesophagitis OR porphyria OR 'propionic acidaemia' OR pruritus OR 'pseudomembranous colitis' OR 'rash skin' OR 'rash' OR 'thrombocytopenia' OR urticaria OR 'vaginal candidiasis' OR 'vaginitis' OR 'valproic acid' OR valproate OR 'vertigo' OR 'vomiting' OR 'vulvovaginal mycotic infection') | 631 |
| **Total PIV and (clinical trials or RCTs) + PIV and safety** | #9 + #10 |  | 1605 |

Abbreviations: AE, adverse event; PIV, pivmecillinam; RCT, randomized controlled trial; SAE, serious adverse event.

## **Supplementary Table 4. Characteristics of Included Studies (N=110)**

| **Study location** | - Europe (n=82) - North America (n=8) - Asia (n=6) - Multiple locations (n=3) - No country information (n=11) |
| --- | --- |
| **Trial description** | - Clinical trials (n=80) - Observational studies (n=25) - Case-control study (n=1) - Integrated literature review (n=1) - Systematic review and meta-analysis (n=1) - No design reported (n=2) |
| **Age of patient population** | - >18 years (n=41) - Adults (18–60 years) only (n=32) - Older adults (>60 years) only (n=4) - Pediatric patients (<18 years) (n=15) - Pediatric and adult patients (n=9) - All age groups (n=5) - No age distribution reported (n=4) |
| **Infection outcomes** | - UTIs including bacteriuria and cystitis (n=66, including 3 bacteriuria during pregnancy) - Outcomes in pregnant patients (n=8) - Acute pyelonephritis (n=7) - Acute exacerbations of chronic bronchitis (n=5) - Enteric fever (n=5) - Acute respiratory tract infection (n=4) - Acute shigellosis (n=3) - Other infections (n=12) |

Abbreviation: UTI, urinary tract infection.

**Supplementary Table 5. Studies not summarized in main tables**

| **Study** | **Key safety observations in subjects treated with mecillinam/pivmecillinam** |
| --- | --- |
| Aaraas et al, 1977 [1] | No side effects |
| Atkins & Talbot, 1987 [2] | Incidence of side effects in 10% (pivmecillinam/pivampicillin) |
| Bailey et al, 1982 [3] | Minimal side effects^a^ |
| Ball et al, 1979 [4] | 3 (12%) with complications of therapy (drug fever [n=2] and hemolysis [n=1; attributed to probenecid]) |
| Barclay et al, 1982 [5] | Well tolerated; discontinuation due to dyspepsia/unpleasant aftertaste |
| Beumer & Sips, 1984 [6] | 1 (3%) discontinuation due to aggravation of bronchoconstriction (pivmecillinam/pivampicillin) |
| Bjerrum et al, 2009 [7] | Minor AEs in 14%, mostly GI |
| Brumfitt & Hamilton-Miller, 1982 [8] | Minor side effects in 6 (14%); 1 (severe headache) caused discontinuation |
| Bukh, 1983 [9] | 1 (3%) discontinuation due to dyspepsia (pivmecillinam/pivampicillin) |
| Clarke et al, 1976 [10] | 1 (13%) discontinuation due to vomiting |
| Damsgaard et al, 1979 [11] | 1 (4%) case of nausea/vomiting |
| Deasy & Lynch, 1980 [12] | No side effects |
| de Louvois & Mulhall, 1983 [13] | No adverse reactions |
| de Louvois et al, 1981 [14] | No adverse reactions |
| Ekberg et al, 1978 [15] | Well tolerated, including in patients with impaired renal function – no discontinuations or dose reductions. Slight GI discomfort in 5 (19%), moderate increase in serum ALT and serum AST in 5 (19%) |
| Ekwall et al, 1980 [16] | 5 (7%) with exanthema (mecillinam probable cause in 1, possible cause in 3). High serum aminotransferases in 23 (32%), all decreased during therapy |
| File & Tan, 1983 [17] | Mecillinam/cefoxitin: mild eosinophilia in 1 (5%). Minimal increase in ALT (1 [5%]) or ALP (1 [5%]). Increase in creatinine (1.5 mg % to 4.0 mg %) thought to be secondary to interstitial nephritis |
| Frimodt-Møller & Ravn, 1979 [18] | Mild pain at injection site in 2 (10%) |
| Frimodt-Møller & Vejlsgaard, 1981 [19] | Upper GI side effects in 3 (25%; pivmecillinam) and 2 (14%; pivmecillinam/pivampicillin). Mild erythematous rash in 1 (7%; pivmecillinam/pivampicillin) |
| Guttmann, 1977 [20] | 1 (2%) discontinuation (mouth ulcers/vaginitis) |
| Hovelius et al, 1985 [21] | Abdominal discomfort/rash in 6% on 3-day course and 3% on 7-day course. No treatment interruptions |
| Igesund & Vorland, 1982 [22] | Rash in 1 (9%; pivmecillinam/pivampicillin) |
| Ishigami, 1977 [23] | Side effects in 3 (3%): throat irritation, chapped lips, headache |
| Jansåker et al, 2018 [24] | Adverse reactions were few and mild (73 [18%]) and primarily seen when ampicillin  was co-administered (69 [95%]) |
| Jodal et al, 1989 [25] | Vaginal discharge leading to discontinuation in 1 (5%) |
| Jusuf et al, 1998 [26] | Nausea in 2 (5%)^a^ |
| Kabir et al, 1984 [27] | No adverse reactions |
| Kalager et al, 1978 [28] | Nausea and vomiting in 2 (7%) |
| Kasholm-Tengve & Bartholdson, 1986 [29] | Treatment-related side effects in 4 (5%) patients treated with combination of ampicillin, mecillinam, and metronidazole (urticaria [n=3] and monilial infection [n=1]) |
| Kurokawa & Fujimura, 1978 [30] | Side effects in 1 (1%): gastric discomfort, not leading to discontinuation |
| Lal et al, 1984 [31] | No side effects |
| Lawson et al, 1983 [32] | Skin rash in 6 (4%) treated with mecillinam alone or in combination with cefoxitin or with ticarcillin and carbenicillin |
| Limson et al, 1982 [33] | Epigastric discomfort in 3 (20%) taking pivmecillinam/pivampicillin. No AEs in patients taking mecillinam alone |
| Nilsen et al, 2016 [34] | Well tolerated; no reported serious AEs |
| Piipo et al, 1985 [35] | Mild GI side effects in 2%^a^ |
| Richards, 1984 [36] | Side effects in 7 (7%) patients on 3-day course, 12 (13%) on 7-day course. Discontinuation in 1 and 2 patients, respectively |
| Salam et al, 1998 [37] | Joint pain/limp in 29%; other varied AEs, not judged related to drug therapy^b^ |
| Saltvedt & Schøyen, 1982 [38] | No AEs |
| Sehested et al, 2017 [39] | 14 (3%) patients treated with either pivmecillinam or amoxicillin-clavulanate switched to parenteral antibiotics due to vomiting (n=7) or ‘other problems’ (n=7)^a^ |
| Siasoco & Uy, 1984 [40] | Minimal side effects, easily tolerated |
| Svarva & Wessel-Aas, 1980 [41] | No side effects observed (patients with severely impaired renal function) |
| Tanphaichitra et al, 1984 [42] | No side effects observed |
| Uylangco et al, 1984 [43] | No side effects observed |
| Verrier-Jones & Asscher, 1975 [44] | Very mild erythematous rash in 1 (3%) |
| Vik et al, 2018 [45] | 1 (0.6%) SAE reported, not related to study drug (alcoholic withdrawal) |
| Ward et al, 1983 [46] | Mecillinam/cefoxitin combination treatment: adverse reactions minimal (mild diarrhea [n=1; 6%], mild reversible elevations in AST levels [n=2; 12%]) |
| Wise et al, 1976 [47] | Slightly elevated AST/ALT in 2 (5%), diarrhea/oral candidiasis in 1 (3%) |

^a^ Based on abstract.

^b^ Study in children with *Shigella* dysentery. Rate of joint pain/limp in comparator group (ciprofloxacin): 23%.

Abbreviations: AE, adverse event; ALP, alkaline phosphatase; ALT, alanine transaminase; AST, aspartate transaminase; GI, gastrointestinal; SAE, serious adverse event.

**Supplementary Table 6. Studies of Mecillinam/Pivmecillinam Used in Combination (Including Fixed-Dose Combination of Pivmecillinam and Pivampicillin)**

| **Study** | **Design and Study Population** | **Treatment Groups^a^** | **Adverse Reactions, n (%)** | | | | | **Discontinued Due to AEs, n (%)** |
| --- | --- | --- | --- | --- | --- | --- | --- | --- |
|  |  |  | **Overall** | **GI Tract Related** | **Skin Related** | **Genitourinary** | **Other** |  |
| **Studies of Pivmecillinam in Free Combination** | | | | | | | | |
| Rotstein et al, 1983 [48] | Prospective study; US  Patients with serious infection due to gram-negative bacillus (pyelonephritis, pneumonia, UTI)  Age range 18–80 y | MEC 10 mg/kg in combination with another β-lactam antibiotic (ampicillin, cephalothin, cefamandole, or cefoxitin) q6h IV for at least 4 d and no longer than 14 d (n=25) | 6 (24.0) | NR | NR | NR | Slight elevation of SGOT levels: 3 (12.0)  Transient leukopenia: 1 (4.0)  Drug fever: 1 (4.0)  Red cell hemolysis: 1 (4.0) | NR |
| Pines et al, 1981 [49] | Prospective study; UK  Patients with lower RTI or UTI  Age range 26–84 y | MEC 400 mg tid + amoxicillin (1.5 g), IM amoxicillin (1.5 g), oral ampicillin (1.5 g, 0.75 g), and cefuroxime (2.25 g) daily for 7 d (n=105) | 3 (2.9) | Diarrhea: 1 (1.0)  Abdominal discomfort: 1 (1.0) | Erythematous rash: 1 (1.0) | NR | NR | 2 (1.9): 1 due to diarrhea, 1 due to erythematous rash |
| King et al, 1983 [50] | Prospective study; US  Patients infected with UTI, sepsis, pneumonia, miscellaneous infections | MEC 10 mg/kg + other β-lactam antibiotics q6h  Patients were followed for 6 wk following cessation of therapy (n=380) | 23 (6.1) | Diarrhea  Nausea  Gastritis with bleeding: 1 (0.3) | Rashes with or without pruritus: 6 (1.6) | Vaginitis | Laboratory abnormalities: 35 (9.2)  Phlebitis  Fever  Arthralgias  Anemia  Thrombocytosis, eosinophilia, and elevations in serum levels of alkaline phosphatase, bilirubin, and AST | 7 (1.8): 6 due to rashes, with or without pruritus and 1 was prompted by leukopenia |
| Bentzen et al, 1975 [51] | Clinical trial; Denmark  Patients having significant bacteriuria with Enterobacteriaceae  Age range 54–95 y | PIV 300 mg qid during Wk 0–2, 150 mg qid during Wk 2–6, 150 mg bid during Wk 6–10 (n=24) | 6 (25.0) | Stomatitis: 1 (4.2)  Loss of appetite, nausea, vomiting: 4 (16.7) | Exanthema: 1 (4.2) | NR | Anorexia: 2 (8.3) | 3 (12.5) |
|  |  | Pivampicillin 700 mg qid during Wk 0–2, 350 mg qid during Wk 2–6, 350 mg bid during Wk 6–10 (n=21) | 9 (42.9) | Nausea and/or vomiting: 4 (19.0)  Stomatitis: 1 (4.8) | Skin rash: 6 (28.6) | NR | Anorexia: 1 (4.8) | 8 (38.1) |
|  |  | PIV 300 mg bid + pivampicillin 700 mg bid during Wk 0–2, PIV 150 mg bid + pivampicillin 350 mg bid during Wk 2–6, PIV 150 mg qd + pivampicillin 350 mg qd during Wk 6–10 (n=25) | 7 (28.0) | Diarrhea: 1 (4.0) | Skin rash: 6 (24.0) | NR | NR | 7 (28.0) |
| **Fixed-Dose Combination of Pivmecillinam + Pivampicillin** | | | | | | | | |
| Holme et al, 1989 [52] | Analysis of carnitine metabolism following short- and long-term treatment; Sweden  UTI  Age range 8 mo to 6 y 5 mo (two adults also reported, but no AE data) | Children: PIV 50–100 mg/d + pivampicillin 62.5–125 mg/d (n=7) | NR | NR | NR | NR | Aggressiveness and nausea: 1 (14.3)  Tiredness and frequent screaming: 1 (14.3)  Mean total serum carnitine concentration fell to 15% (7%–27%) of pretreatment values: 7 (100.0)  Muscle carnitine concentrations in two girls treated with the antibiotics for 22 mo and 30 mo were only 10% of the mean reference value | 2 (28.6): 1 aggressive-ness and nausea and 1 tiredness and frequent screaming |
| Holme et al, 1992 [53] | Observational study; Sweden  Children with dilated vesicoureteral reflux or recurrent pyelonephritis  Age range 2–9.5 y | PIV and pivampicillin 250–500 μmol/d for >12 mo (n=17) | NR | NR | NR | NR | Reduction of the free carnitine concentration in serum and muscle to less than 10% of the mean reference value  Tired and whiny with pain in the legs and limited walking: 1 (5.9)  Clumsy with poor endurance: 1 (5.9) | **During prophylaxis:**  Allergic reaction: 1 (5.9)  Tired and whiny with pain in the legs and limited walking: 1 (5.9)  Clumsy with poor endurance: 1 (5.9)  Cessation due to carnitine depletion: 9 |
| Svenungsson et al, 1990 [54] | Double-blind RCT; Sweden  Carriers of nontyphi *Salmonella* species for  10-21 wk  Age range 16–78 y | Pivampicillin 0.25 g and PIV 0.20 g  Two tablets tid for 4 wk (n=16) | NR | Nausea: 4 (25.0) | Pruritus: 3 (18.8)  Exanthema: 6 (37.5) | NR | Fever: 1 (6.3) | 7 (43.8): exanthema, nausea, pruritus, fever |
| Jernelius et al, 1988 [55] | Double-blind RCT; Sweden  Acute pyelonephritis  Age range 16–81 y | Group A: pivampicillin 0.25 g and PIV 0.20 g, two tablets tid days  1–7 followed by placebo (one tablet tid, days 8–21) (n=38) | **1-wk Tx:**  With AEs: 5 (13.2) | **1-wk Tx:**  Diarrhea: 2 (5.3) | **1-wk Tx:**  Exanthema/skin rash: 1 (2.6)  Urticaria: 1 (2.6) | NR | **1-wk Tx:**  Photophobia: 1 (2.6)  Mild increase in amino-transferase levels: 2 (5.3) | **1-wk Tx:**  2 (5.3) |
|  |  | Group B: pivampicillin 0.25 g and PIV 0.20 g, two tablets tid days 1–7, then reduced to one tablet tid for days 8–21 (n=39) | **3-wk Tx:**  With AEs: 8 (20.5) | **3-wk Tx:**  Diarrhea: 3 (7.7)  Dyspepsia: 2 (5.1)  Loose stools: 1 (2.6)  Nausea/ vomiting: 5 (12.8) | **3-wk Tx:**  Urticaria: 1 (2.6) |  | **3-wk Tx:**  Mild increase in amino-transferase levels: 1 (2.6) | **3-wk Tx:**  4 (10.3) |
| Geerdsen et al, 1983 [56] | Double-blind RCT; Denmark  UTI  Age range 32–93 y | PIV 100 mg and pivampicillin 125 mg, two tablets bid for 10 d (n=44) | NR | Anorexia: 2 (4.5)  Loose stools: 2 (4.5)  Eructation in connection with tablet intake: 1 (2.3) | NR | NR | NR | NR |
|  |  | PIV 200 mg, two tablets bid for 10 d (n=33) | NR | Diarrhea: 1 (3.0) | NR | NR | NR | 1 (3.0): patient with an esophageal stricture and difficulty swallowing |
| Eriksson et al, 1986 [57] | RCT; Sweden  Acute pyelonephritis  Age range 15–86 y | Pivampicillin 0.25 g and PIV 0.20 g tid for 14 d (n=43) | 15 (34.9) | Nausea: 3 (7.0)  Esophagitis: 1 (2.3) | Exanthema: 7 (16.3)  Pruritus: 2 (4.7) | NR | Eosinophilia: 3 (7.0)  High ALT, AST levels: 2 (4.7)  Headache: 3 (7.0) | 4 (9.3) |
|  |  | PIV 0.7 g tid for 14 d (n=50) | 6 (12.0) | Nausea: 1 (2.0) | Exanthema: 1 (2.0) | NR | Eosinophilia: 2 (4.0)  Lymphocytosis: 2 (4.0)  High ALT, AST levels: 1 (2.0)  Headache: 2 (4.0) | NR |
| Johansen et al, 1999 [58] | Single-blind RCT; Denmark  Patients with COPD or CF  Age range 1–40 y | Pivampicillin 50 mg/kg/d and PIV 40 mg/kg/d (n=37) | 17 (45.9) | Abdominal pain: 5 (13.5)  Constipation: 1 (2.7)  Diarrhea: 1 (2.7)  Nausea/ vomiting: 7 (18.9) | NR | NR | Headache: 1 (2.7) | (Tx interruption) 7 (18.9) |
|  |  | PIV 40 mg/kg/d (n=34) | 11 (32.4) | Abdominal pain: 4 (11.8)  Diarrhea: 7 (20.6)  Nausea/ vomiting: 4 (11.8) | Skin rash: 1 (2.9) | NR | Fatigue: 1 (2.9) | (Tx interruption) 4 (11.8) |
| Beatson et al, 1985 [59] | Single-blind multicenter trial; UK  Upper or lower RTI  Age range 10–85 y | PIV 200 mg and pivampicillin 250 mg bid for 7 d (n=205) | 19 (9.3) | GI disturbance: 16 (7.8) | Rash: 2 (1.0) | NR | Headache: 1 (0.5)  Other: 3 (1.5) | NR |
|  |  | Combination of tetracycline hydrochloride 115.4 mg, chlortetracycline hydrochloride 115.4 mg, and demeclocycline hydrochloride 69.2 mg bid for 7 d (n=203) | 35 (17.5) | GI disturbance: 16 (7.9)  Abdominal cramps: 4 (2.0)  Vomiting: 3 (1.5) | NR | NR | Headache: 5 (2.5)  Tiredness/ lethargy/ malaise: 5 (2.5)  Giddiness/ dizziness: 1 (0.5)  Sore/dry mouth: 2 (1.0)  Others: 5 (2.5) | 6 (3.0) |
| Shenderey et al, 1985 [60] | Single-blind RCT, UK  Acute RTI  Age range 11–86 y | PIV 200 mg and pivampicillin 250 mg bid for 7 d (n=174) | 17 (9.8) | Upper gastric discomfort/ nausea/ indigestion: 3 (1.7)  Loose stools/ diarrhea: 4 (2.3) | Rash: 1 (0.6)  Prickly sensation under skin: 1 (0.6) | NR | Headache: 1 (0.6)  Tiredness/ lethargy/ weakness: 4 (2.3)  Dizziness: 2 (1.1)  Sore mouth/sore throat: 1 (0.6)  Leg pains: 1 (0.6) | 4 (2.3): upper gastric discomfort/ nausea/ indigestion: 2 (1.1); loose stools/ diarrhea: 1 (0.6); sore mouth/sore throat: 1 (0.6) |
|  |  | Amoxicillin 250 mg tid for 7 d (n=175) | 15 (8.6) | Upper gastric discomfort/ nausea/ indigestion: 4 (2.3)  Loose stools/ diarrhea: 8 (4.6)  Anorexia: 1 (0.6)  Anal irritation: 1 (0.6) | NR | NR | Headache: 1 (0.6) | 2 (1.1): both loose stools/ diarrhea |
| McGhie et al, 1986 [61] | Single-blind multicenter study; UK  Acute bronchitis or acute exacerbations of chronic bronchitis  Age range 18–70 y | PIV 200 mg and pivampicillin 250 mg, two tablets bid for 7 d (acute bronchitis) or 10 d (chronic bronchitis) (n=218) | 26 (11.9) | Upper GI: 10 (0.5)  Lower GI: 4 (1.8) | Rash: 3 (1.3)  Angioneurotic edema: 1 (0.5) | NR | Dizziness: 3 (1.4)  Headache: 5 (2.3)  Tired: 1 (0.5)  Sore throat: 1 (0.5)  Thirst: 1 (0.5) | 5 (2.3) |
|  |  | Amoxicillin 250 mg and clavulanic acid 125 mg, one tablet tid for 7 d (acute bronchitis) or 10 d (chronic bronchitis) (n=210) | 26 (12.4) | Upper GI: 6 (2.9)  Lower GI: 8 (3.8) | Rash: 3 (1.4) | NR | Dizziness: 3 (1.4)  Tired: 2 (1.0)  Joint pain: 3 (1.4)  Tremor: 1 (0.5)  Pruritus vulvae: 1 (0.5)  Generally unwell: 1 (0.5)  Green urine: 1 (0.5) | 7 (3.3) |
| O’Dowd et al, 1984 [62] | Open-label RCT; UK  Acute UTI  Age range 20–81 y | PIV 200 mg and pivampicillin 250 mg bid for 5 d (n=29) | 8 (27.6) | Vomiting, abdominal pain (n not specified) | NR | Thrush (n not specified) | NR | 1 (3.4) |
|  |  | Amoxicillin 250 mg plus clavulanate 125 mg tid for 5 d (n=29) | 5 (17.2) | Vomiting, abdominal pain (n not specified) | NR | Thrush (n not specified) | NR | 1 (3.4) |
| Iosif et al, 1983 [63] | RCT; Sweden  UTI  Age range 21–79 y | PIV 125 mg and pivmecillinam 100 mg, two tablets tid for 10 d (n=40) | 7 (17.5) | Diarrhea: 2 (5.0)  Nausea: 1 (2.5) | Pruritus: 1 (2.5)  Exanthema: 2 (5.0) | NR | Hematological abnormalities: 5 (12.5) | 2 (5.0) |
|  |  | Trimethoprim 80 mg and sulfamethoxazole 400 mg, two tablets bid for 10 d (n=36) | 15 (41.7) | Nausea: 2 (5.6) | Pruritus: 2 (5.6)  Exanthema: 8 (22.2) | NR | Hematological abnormalities: 9 (25)  Liver function parameters increased: 1 (2.8)  Headache: 1 (2.8) | 8 (22.2) |
| Ravn, 1981 [64] | Open RCT; Denmark  Hospital inpatients with complicated UTI  Age range 34–82 y | PIV 200 mg and pivampicillin 250 mg, two tablets bid for 10 d (n=23) | 4 (17.4) | Severe diarrhea: 1 (4.3)  Mild loose stools: 1 (4.3) | Transient urticarial rash: 1 (4.3) | NR | Mild colic: 1 (4.3)  Moderate anorexia and severe colic: 1 (4.3) | NR |
|  |  | Trimethoprim 160 mg and sulfamethoxazole 800 mg, two tablets bid for 10 d (n=19) | 2 (10.5) | NR | NR | NR | Moderate anorexia: 1 (5.3)  Mild eosinophilia: 1 (5.3) | NR |
| Wallace et al, 1985 [65] | Single-blind RCT, UK  Patients with signs and symptoms of upper or lower RTI  Age range 10–76 y | PIV 200 mg and pivampicillin 250 mg bid for 7 d (n=160) | 19 (11.9) | GI upset: 7 (4.4)  Black stools: 1 (0.6) | Rash: 2 (1.3) | NR | Headache: 1 (0.6)  Mouth ulcer: 1 (0.6)  Lethargy and weakness (combined): 2 (1.3)  Vertigo and dizziness (combined): 1 (0.6)  Sore mouth/tongue: 2 (1.3)  Dry mouth: 2 (1.3)  Cough: 1 (0.6) | 2 (1.3) |
|  |  | Trimethoprim 160 mg and sulfamethoxazole 800 mg bid for 7 d (n=158) | 24 (15.2) | GI upset: 13 (8.2) | Rash: 3 (1.9) | NR | Headache: 2 (1.3)  Lethargy and weakness (combined): 1 (0.6)  Vertigo and dizziness (combined): 4 (2.5)  Sore mouth/tongue: 2 (1.3)  Dry mouth: 3 (1.9)  Palpitations: 1 (0.6)  Paresthesia/pain in foot: 1 (0.6)  Feeling hot after tablets: 1 (0.6) | 4 (2.5) |
| Holmquist & Lundgren, 1984 [66] | RCT; Sweden  Patients undergoing transurethral prostate resection  Age range 50–84 y | PIV 100 mg and pivampicillin 125 mg, two tablets tid for 10 d (n=77) | 5 (6.5) | Mild loose stool: 1 (1.2) | Rash: 2 (2.5)  Pruritus: 1 (1.2) | NR | Mild anorexia: 1 (1.2) | 1 (1.2): rash |
|  |  | Trimethoprim 80 mg and sulfamethoxazole 400 mg, two tablets bid for 10 d (n=74) | 2 (2.7) | Mild diarrhea: 1 (1.4) |  | NR | Vertigo and anorexia: 1 (1.4) | NR |
| Cronberg et al, 1995 [67] | Double-blind RCT; Sweden  Severe pneumonia or pyelonephritis  Mean age 61 y | Pivampicillin 600 mg + PIV 300 mg IV, followed by pivampicillin 250 mg + PIV 200 mg oral for 14 d (n=144) | 32 (22.2) | Nausea, vomiting, epigastric pain: 11 (7.6)  Diarrhea: 4 (2.8)  Stomatitis, vulvovaginitis, or urethritis: 3 (2.1) | Exanthema: 12 (8.3) | NR | Thrombo-phlebitis: 2 (1.4) | 16 (11.1): 1 due to diarrhea |
|  |  | Cefotaxime 1000 mg IV, followed by cephalosporin 400 mg (capsules) for 14 d (n=149) | 41 (27.5) | Nausea, vomiting, epigastric pain: 10 (6.7)  Diarrhea: 11 (7.4)  Stomatitis, vulvovaginitis, or urethritis: 6 (4.0) | Exanthema: 6 (4.0) | NR | Thrombo-phlebitis: 4 (2.7)  Other events: 4 (2.7) | 16 (10.7): 6 due to diarrhea |
| Grabe et al, 1986 [68] | RCT; Sweden  Transurethral resection for hyperplasia or cancer of the prostate  Age range 51–94 y | Part 1: pivampicillin 250 mg + PIV 200 mg bid for ≤7 d (n=60)  Part 2: pivampicillin 500 mg + PIV 400 mg bid for ≤7 d (n=69) | 3 (2.3) | Diarrhea: 2 (1.6) | Exanthema: 1 (0.8) | NR | NR | 2 (1.6) (Tx interruption due to diarrhea) |
|  |  | Cefotaxime 1 g qd (n=62, part 1; n=70, part 2) | 0 | NR | NR | NR | NR | NR |
| Spindler et al, 1985 [69] | RCT, abstract  Upper or lower RTIs  N=424 for both groups combined | PIV 200 mg and pivampicillin 250 mg bid for 7 d | 23 (10.8) | NR | NR | NR | NR | 4 (1.9) |
|  |  | Cephalexin 500 mg tid for 7 d | 30 (14.4) | NR | NR | NR | NR | 5 (2.4) |

^a^ n represents size of safety population.

Abbreviations: AE, adverse event; ALT, alanine aminotransferase; AST, aspartate aminotransferase; bid, twice daily; CF, cystic fibrosis; COPD, chronic obstructive pulmonary disease; d, days; GI, gastrointestinal; IM, intramuscular; IV, intravenous; MEC, mecillinam; mo, months; NR, not reported; PIV, pivmecillinam; q6h, every 6 hours; qd, once daily; qid, four times daily; RCT, randomized controlled trial; RTI, respiratory tract infection; SGOT, serum glutamic-oxaloacetic transaminase; tid, three times daily; Tx, treatment; UTI, urinary tract infection; wk, weeks; y, years.

**References**

1. Aaraas I, Skarsten KW, Neess HC. Pivmecillinam in the treatment of post-operative bacteriuria in gynecological patients. A double-blind comparison with pivmecillinam and pivampicillin. J Antimicrob Chemother **1977**; 3(3): 227–32.

2. Atkins MJ, Talbot DJ. A comparison of pivmecillinam/pivampicillin and amoxycillin in acute exacerbations of chronic bronchitis. J Int Med Res **1987**; 15(2): 115–20.

3. Bailey RR, Peddie B, Chambers PF, et al. Single dose doxycycline, cefuroxime and pivmecillinam for treatment of bacterial cystitis. N Z Med J **1982**; 95(717): 699–700.

4. Ball AP, Farrell ID, Gillett AP, Geddes AM, Clarke PD, Ellis CJ. Enteric fever in Birmingham: clinical features, laboratory investigation and comparison of treatment with pivmecillinam and co-trimoxazole J Infect **1979**; 1(4): 353–65.

5. Barclay RP, Mejlhede A, Nilsson LB. Long-term, low-dose treatment with pivmecillinam alone and in combination with pivampicillin in patients prone to recurrent bacteriuria. Curr Med Res Opin **1982**; 8(2): 82–8.

6. Beumer HM, Sips AP. Miraxid versus Augmentin in exacerbations of chronic bronchitis Drugs Exp Clin Res **1984**; 10(5): 351–6.

7. Bjerrum L, Gahrn-Hansen B, Grinsted P. Pivmecillinam versus sulfamethizole for short-term treatment of uncomplicated acute cystitis in general practice: a randomized controlled trial. Scand J Prim Health Care **2009**; 27(1): 6–11.

8. Brumfitt W, Hamilton-Miller JM. Pivmecillinam in complicated urinary infections failing to respond to conventional therapy. Infection **1982**; 10(3): 149–52.

9. Bukh N. Double-blind comparison of pivmecillinam plus pivampicillin ('Miraxid') with pivampicillin alone in chronic bronchitis: a Danish multi-centre study. Pharmatherapeutica **1983**; 3(6): 422–8.

10. Clarke PD, Geddes AM, McGhie D, Wall JC. Mecillinam: a new antibiotic for enteric fever. Br Med J **1976**; 2(6026): 14–5.

11. Damsgaard T, Jacobsen J, Korner B, Tybring L. Pivmecillinam and trimethoprim/sulfamethoxazole in the treatment of bacteriuria. A bacteriological and pharmacokinetic study. J Antimicrob Chemother **1979**; 5(3): 267–74.

12. Deasy PF, Lynch K. Pivmecillinam in the treatment of recurrent urinary infection in girls. Ir Med J **1980**; 73(10): 388–9.

13. de Louvois J, Mulhall A. Efficacy, pharmacology, and safety of amdinocillin in treatment of neonates. Am J Med **1983**; 75(2A): 119–24.

14. de Louvois J, Mulhall A, Hurley R. Mecillinam (Selexidin) in the treatment of neonates. J Perinat Med **1981**; 9(2): 87–95.

15. Ekberg M, Denneberg T, Larsson S, Juhlin I. Pharmacokinetic and therapeutic studies of pivmecillinam in patients with normal and impaired renal function. Scand J Infect Dis **1978**; 10(2): 127–33.

16. Ekwall E, Scheja A, Cronberg S, et al. Mecillinam and ampicillin separately or combined in gram-negative septicemia. Infection **1980**; 8(1): 37–40.

17. File TM, Jr., Tan JS. Amdinocillin plus cefoxitin versus cefoxitin alone in therapy of mixed soft tissue infections (including diabetic foot infections). Am J Med **1983**; 75(2A): 100–5.

18. Frimodt-Møller N, Ravn TJ. Mecillinam in urinary tract infections and in septicaemia. Infection **1979**; 7(1): 35–7.

19. Frimodt-Møller C, Vejlsgaard R. Pivmecillinam plus pivampicillin in complicated urinary tract infection. Double-blind comparison of the combination pivmecillinam/pivampicillin and pivmecillinam alone in patients with urinary tract infection. J Int Med Res **1981**; 9(4): 283–7.

20. Guttmann D. A comparison of pivmecillinam and cotrimoxazole in the treatment of simple cystitis in general practice. J Antimicrob Chemother **1977**; 3(Suppl B): 137–40.

21. Hovelius B, Mårdh PA, Nygaard-Pedersen L, Wathne B. Nalidixic acid and pivmecillinam for treatment of acute lower urinary tract infections. Scand J Prim Health Care **1985**; 3(4): 227–32.

22. Igesund A, Vorland L. A fixed combination of pivmecillinam and pivampicillin in complicated urinary tract infections. A double-blind comparison with pivmecillinam. Scand J Infect Dis **1982**; 14(2): 159–60.

23. Ishigami J. Clinical evaluation of pivmecillinam in acute simple cystitis: a comparative study with amoxycillin by a randomized double-blind technique. Journal of Antimicrobial Chemotherapy **1977**; 3(suppl_B): 129−35.

24. Jansåker F, Frimodt-Møller N, Benfield TL, Knudsen JD. Mecillinam for the treatment of acute pyelonephritis and bacteremia caused by Enterobacteriaceae: a literature review. Infect Drug Resist **2018**; 11: 761–71.

25. Jodal U, Larsson P, Hansson S, Bauer CA. Pivmecillinam in long-term prophylaxis to girls with recurrent urinary tract infection. Scand J Infect Dis **1989**; 21(3): 299–302.

26. Jusuf H, Sudjana P. Mecillinam for the treatment of Typhoid fever. Medical Journal of Indonesia **1998**; 7(1): 195.

27. Kabir I, Rahaman MM, Ahmed SM, Akhter SQ, Butler T. Comparative efficacies of pivmecillinam and ampicillin in acute shigellosis. Antimicrob Agents Chemother **1984**; 25(5): 643–5.

28. Kalager T, Bøe E, Digranes A, Høisaether P, Solberg CO. Pivmecillinam treatment of chronic urinary tract infection. Infection **1978**; 6(1): 21–2.

29. Kasholm-Tengve B, Bartholdson P. Antibiotics in acute abdominal surgery. A clinical trial comparing the combination of ampicillin, mecillinam and metronidazole with cefoxitin alone. Acta Chir Scand **1986**; 152: 267–72.

30. Kurokawa K, Fujimura N. Comparative controlled clinical trial of pivmecillinam and nalidixic acid in patients with acute simple urinary tract infections. Jpn J Antibiot **1978**; 31(12): 701–11.

31. Lal S, McGhie D, Kerfoot P. A comparison of pivmecillinam/pivampicillin and co-trimoxazole in hospitalized patients with acute exacerbations of chronic bronchitis. J Antimicrob Chemother **1984**; 14(2): 179–84.

32. Lawson RD, Estey EH, Bodey GP. Amdinocillin: use alone or in combination with cefoxitin or carbenicillin-ticarcillin. Am J Med **1983**; 75(2A): 113–8.

33. Limson BM, Mendoza MT, Liwanag E, Christensen OE, Menday P. Randomised, comparative trial of mecillinam, mecillinam/ampicillin and chloramphenicol in the treatment of enteric fever. J Antimicrob Chemother **1982**; 9(5): 405–10.

34. Nilsen E, Aasterød M, Hustad PS, Olsen AO. Mecillinam against genital *Chlamydia trachomatis* infection: a small-scale proof-of-concept study shows a low cure rate. J Antimicrob Chemother **2016**; 71(8): 2270–2.

35. Piipo T, Pyykonen M, Pitkajarvi T. Pivmecillinam in the treatment of acute cystitis. Current Therapeutic Research **1985**; 37(2): 184–90.

36. Richards HH. Comparative efficacy of 3-day and 7-day chemotherapy with twice-daily pivmecillinam in urinary tract infections seen in general practice. Curr Med Res Opin **1984**; 9(3): 197–203.

37. Salam MA, Dhar U, Khan WA, Bennish ML. Randomised comparison of ciprofloxacin suspension and pivmecillinam for childhood shigellosis. Lancet **1998**; 352(9127): 522–7.

38. Saltvedt EP, Schøyen R. Pivmecillinam plus pivampicillin in urinary tract infections. A double-blind comparison with pivmecillinam alone in hospitalized patients. Infection **1982**; 10(1): 21–2.

39. Sehested LT, K. K, Winding L, et al. Oral antibiotic treatment for pyelonephritis is safe and effective if there is a formalised contact to the department of paediatrics. Pediatr Nephrol **2017**; 32(9): 1645.

40. Siasoco R, Uy ND. Pivmecillinam in the treatment of acute lower urinary tract infections. Philipp J Intern Med **1984**; 22(1): 19–22.

41. Svarva PL, Wessel-Aas T. Serum levels of mecillinam in patients with severely impaired renal function. Scand J Infect Dis **1980**; 12(4): 303–5.

42. Tanphaichitra D, Srimuang S, Chiaprasittigul P, Menday P, Christensen OE. The combination of pivmecillinam and pivampicillin in the treatment of enteric fever. Infection **1984**; 12(6): 381–3.

43. Uylangco C, Santiago L, Pescante M, Menday P, Christensen O. Pivmecillinam, co-trimoxazole and oral mecillinam in gastroenteritis due to *Vibrio* spp. J Antimicrob Chemother **1984**; 13(2): 171–5.

44. Verrier Jones ER, AW. A. Treatment of recurrent bacteriuria with pivmecillinam (FL 1039). J Antimicrob Chemother **1975**; 1(2): 193–6.

45. Vik I, Bollestad M, Grude N, et al. Ibuprofen versus pivmecillinam for uncomplicated urinary tract infection in women-A double-blind, randomized non-inferiority trial. PLoS Med **2018**; 15(5): e1002569.

46. Ward TT, Amon MB, Krause LK. Combination amdinocillin and cefoxitin therapy of multiply-resistant *Serratia marcescens* urinary tract infections. Am J Med **1983**; 75(2A): 85–9.

47. Wise R, Reeves DS, Symonds JM, Wilkinson PJ. A clinical investigation of pivmecillinam. A novel beta-lactam antibiotic in the treatment of urinary tract infections. Chemotherapy **1976**; 22(5): 335–9.

48. Rotstein C, Farrar WE, Jr. Amdinocillin in combination with beta-lactam antibiotics for treatment of serious gram-negative infections. Am J Med **1983**; 75(2A): 96–9.

49. Pines A, Raafat H, Khorasani MH, Sharma JC, Menday AP. An evaluation of parenteral mecillinam in a chest hospital. Curr Med Res Opin **1981**; 7(3): 137–41.

50. King JW, Beam TR, Jr., Neu HC, Smith LG. Systemic infections treated with amdinocillin in combination with other beta-lactam antibiotics. Am J Med **1983**; 75(2A): 90–5.

51. Bentzen AJ, Vejlsgaard R, Jacobsen J, Tybring L. Clinical evaluation of a novel beta-lactam antibiotic: pivmecillinam (FL 1039). Infection **1975**; 3(3): 154–60.

52. Holme E, Greter J, Jacobson CE, et al. Carnitine deficiency induced by pivampicillin and pivmecillinam therapy. Lancet **1989**; 2(8661): 469–73.

53. Holme E, Jodal U, Linstedt S, Nordin I. Effects of pivalic acid-containing prodrugs on carnitine homeostasis and on response to fasting in children. Scand J Clin Lab Invest **1992**; 52(5): 361–72.

54. Svenungsson B, Ekwall E, Hansson HB. Efficacy of the combination pivampicillin/pivmecillinam compared to placebo in the treatment of convalescent carriers of nontyphi *Salmonella*. Infection **1990**; 18(3): 163–5.

55. Jernelius H, Zbornik J, Bauer CA. One or three weeks' treatment of acute pyelonephritis? A double-blind comparison, using a fixed combination of pivampicillin plus pivmecillinam. Acta Med Scand **1988**; 223(5): 469–77.

56. Geerdsen J, Gefke K, Hansen HL. A fixed combination of pivmecillinam and pivampicillin in complicated urinary tract infections. Double-blind comparison with pivmecillinam alone in hospitalized patients. Scand J Infect Dis **1983**; 15(2): 195–9.

57. Eriksson S, Zbornik J, Dahnsjö H, et al. The combination of pivampicillin and pivmecillinam versus pivampicillin alone in the treatment of acute pyelonephritis. Scand J Infect Dis **1986**; 18(5): 431–8.

58. Johansen HK, Børch K, Espersen F, Koch C, Høiby N. Randomised trial of pivampicillin plus pivmecillinam vs. pivampicillin in children and young adults with chronic obstructive pulmonary disease and infection with *Haemophilus influenzae*. Curr Med Res Opin **1999**; 15(4): 300–9.

59. Beatson JM, Marsh BT, Talbot DJ. A clinical comparison of pivmecillinam plus pivampicillin (Miraxid) and a triple tetracycline combination (Deteclo) in respiratory infections treated in general practice. J Int Med Res **1985**; 13(4): 197–202.

60. Shenderey K, Marsh BT, Talbot DJ. A multi-centre general practice comparison of a fixed-dose combination of pivmecillinam plus pivampicillin with amoxycillin in respiratory tract infections. Pharmatherapeutica **1985**; 4(5): 300–5.

61. McGhie D, Kerfoot P, Talbot DJ. A comparative study of Miraxid (pivmecillinam plus pivampicillin) and Augmentin (amoxycillin plus clavulanic acid) in the treatment of lower respiratory tract infections in general practice. J Int Med Res **1986**; 14(5): 254–60.

62. O'Dowd TC, Ribeiro CD, Smail JE, Menday AP, Howells CH. A comparative trial of pivmecillinam/pivampicillin and amoxycillin/clavulanate in the therapy of urinary tract infection in a general practice population. Curr Med Res Opin **1984**; 9(5): 310–5.

63. Iosif CS, Laurin J, Möller EB, Bauer CA. Treatment of urinary tract infections in gynecological patients. A randomized study comparing pivampicillin-pivmecillinam with trimethoprim-sulphamethoxazole. Acta Obstet Gynecol Scand **1983**; 62(5): 515–8.

64. Ravn TJ. Pivmecillinam plus pivampicillin in urinary tract infections: a randomized, open comparison with co-trimoxazole in hospitalized patients. Pharmatherapeutica **1981**; 2(9): 581-6.

65. Wallace RB, Marsh BT, Talbot DJ. A multi-centre general practice clinical evaluation of pivmecillinam plus pivampicillin ('Miraxid') and co-trimoxazole ('Septrin') in respiratory tract infections. Curr Med Res Opin **1985**; 9(10): 659–65.

66. Holmquist B, Lundgren R. Pivmecillinam plus pivampicillin versus co-trimoxazole in patients undergoing transurethral prostate resection. Pharmatherapeutica **1984**; 3(10): 686–91.

67. Cronberg S, Banke S, Bruno AM, et al. Ampicillin plus mecillinam vs. cefotaxime/cefadroxil treatment of patients with severe pneumonia or pyelonephritis: a double-blind multicentre study evaluated by intention-to-treat analysis. Scand J Infect Dis **1995**; 27(5): 463–8.

68. Grabe M, Forsgren A, Hellsten S. The effectiveness of a short perioperative course with pivampicillin/pivmecillinam in transurethral prostatic resection: clinical results. Scand J Infect Dis **1986**; 18(6): 567–73.

69. Spindler JJ, Marsh BT, Talbot DJ. Respiratory infections in general practice. A clinical comparison of pivmecillinam-plus-pivampicillin(Miraxid) and cephalexin(Ceporex). Clinical Trials Journal **1985**; 22(4): 293–9.
